# ActRIIA and BMPRII Type II BMP Receptor Subunits Selectively Required for Smad4-Independent BMP7-Evoked Chemotaxis

**DOI:** 10.1371/journal.pone.0008198

**Published:** 2009-12-08

**Authors:** Jeanette C. Perron, Jane Dodd

**Affiliations:** Departments of Physiology and Cellular Biophysics and Neuroscience, Columbia University, New York, New York, United States of America; National Institutes of Health (NIH), United States of America

## Abstract

Bone morphogenetic protein (BMP)-evoked reorientation and chemotaxis of cells occurs with rapid onset and involves events local to the cell membrane. The signaling pathways underlying these rapid processes likely diverge from those mediating classical transcriptional responses to BMPs but it remains unclear how BMP receptors are utilized to generate distinct intracellular mechanisms. We show that BMP7-evoked chemotaxis of monocytic cells depends on the activity of canonical type II BMP receptors. Although the three canonical type II BMP receptors are expressed in monocytic cells, inhibition of receptor subunit expression by RNAi reveals that ActRIIA and BMPRII, but not ActRIIB, are each essential for BMP7-evoked chemotaxis but not required individually for BMP-mediated induction. Furthermore, the chemotactic response to BMP7 does not involve canonical Smad4-dependent signaling but acts through PI3K-dependent signaling, illustrating selective activation of distinct intracellular events through differential engagement of receptors. We suggest a model of a BMP receptor complex in which the coordinated activity of ActRIIA and BMPRII receptor subunits selectively mediates the chemotactic response to BMP7.

## Introduction

Many growth factors originally characterized for their trophic functions, involving transcriptional activity in target cells, appear also to evoke spatially restricted, tropic events which depend on short latency local regulation of cytoskeletal dynamics [1], [2]. Both trophic and tropic responses can occur in the same cells, raising the question of how the same stimulus activates distinct intracellular signaling programs.

Bone morphogenetic proteins (BMPs) represent such a class of signaling molecules. Classical BMP-evoked gene induction and cellular differentiation occurs widely [3] and the canonical receptors and downstream signaling pathways that give rise to these trophic events have been described in detail [4], [5]. Tropic responses to BMPs include chemotaxis, described in many cell types and tissues [6]–[10], and reorientation of neuronal growth cones [11]–[13], both events result from localized changes in membrane and cytoskeletal organization [14], [15]. However, although evidence is emerging for an array of non-canonical BMP signaling mechanisms [13], [16]–[18], it remains unclear how BMPs initiate local cytoskeletal signaling, whether components of the inductive signaling pathway also mediate tropic signaling and how downstream signaling choice is regulated.

Evidence from *in vitro* assays on primary migrating cells or embryonic spinal neurons suggests that divergence of chemotropic and inductive signaling responses to BMPs, within the same cells, occurs at the receptor level. Thus, of the BMPs with neural inducing capacity, only a subset is active in growth cone orientation [11], [12]. Moreover, BMP7 shows different potencies in chemotropic versus induction assays [7], [11]. Classically, BMP signal transduction is initiated by dimers of BMP binding to a tetrameric receptor complex comprising one pair each of type I (ALK2, BMPRIA or BMPRIB) and type II (ActRIIA, ActRIIB or BMPRII) BMP receptor subunits [5], [19]. The potential for variety in the composition of receptor complexes raises the possibility that a subset of BMPs selectively recruit individual receptor subunits that drive chemotropic responses.

There is growing evidence for divergent mechanisms downstream of receptor activation by BMPs. BMP-mediated induction of gene expression depends on long exposure time, whereas BMP7 acts within minutes to elicit chemotaxis or growth cone collapse [7], [11], suggesting that the underlying intracellular mechanism activating chemotaxis does not rely on transcription. Divergence from the canonical intracellular signaling pathway may lie somewhere along the BMP-evoked Smad signaling cascade, which includes receptor-regulated Smads (R-Smads) specific to BMPs (Smad1, Smad5 and Smad8) and the co-Smad, Smad4 [5], [20], or might occur independent of Smad-mediated signaling in direct response to receptor activation. Recently, R-Smad-independent mechanisms of BMP signal transduction have been shown to regulate transcription downstream of receptor activation [21], [22] but have also been implicated in non-transcriptional mechanisms [4], [23]. In particular, phosphoinositide 3-kinase (PI3K) and LIM kinase 1 (LIMK1), regulators of cytoskeletal dynamics, have both been shown to associate with type II BMP receptor subunits [24], [25], providing a link between BMP receptor activation and cytoskeletal signaling. Activation of PI3K-dependent signaling by BMPs has been implicated in the migration of chondrosarcoma and other cells [17], [18]. Moreover, the identification of PI3K as a common and critical target of other, non-BMP, chemotactic factors [26]–[29], positions this kinase as a potential direct target of BMPs in chemotaxis and other BMP-dependent chemotropic activities.

We have used the chemotaxis of monocytic cells as a model cell system in which to dissect the differential signaling mechanisms underlying chemotactic and inductive responses to a given BMP. Because of the link between type II receptor subunits and the cytoskeleton, we have focused our attention on the potential role of type II BMP receptor subunits in chemotaxis and provide evidence for a model in which ActRIIA and BMPRII subunits, acting in unique combination, possibly as a heterodimer pair, are key players in the chemotactic response to BMP7. Other type II receptor subunit homo- or heterodimer combinations, involving ActRIIB, do not appear to support and are not essential to the chemotactic response. The selective dependence on individual type II BMP receptors in chemotaxis is not exhibited by the inductive activities of BMP7. Moreover, although the classical signaling pathway involving Smads is activated by BMP7 in these cells and results in gene induction, we show that BMP7-evoked chemotaxis is independent of the Smad4-mediated signaling cascade and depends on PI3K activity. Our results support a model in which the selective engagement of a receptor complex containing both ActRIIA and BMPRII subunits by chemotropic BMPs, typified by BMP7, directs a divergent intracellular pathway towards rapid activation of local cytoskeletal dynamics.

## Results

### BMP7 Stimulates a Chemotactic Response in WEHI 274.1 Cells

**Figure 1:**
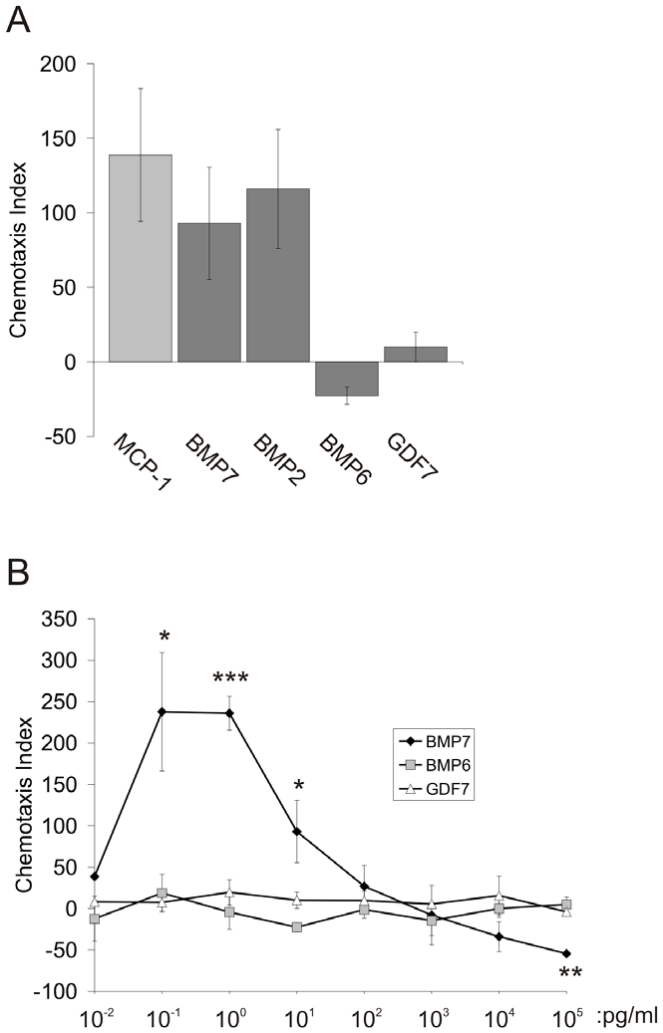
Chemotaxis of monocytic cells in response to a panel of BMPs. A: WEHI 274.1 cell migration through transwell chamber filters in response to 10 pg/ml BMP7, BMP2, BMP6 or GDF7 in the lower chamber. For comparison, cells were also stimulated with MCP-1 (100 ng/ml). The chemotaxis index (CI) is shown as mean +/− SEM. MCP-1 (CI = 139+/−45, n = 4); BMP7 (CI = 93+/−38, n = 5); BMP2 (CI = 116+/−40, n = 4); BMP6 (CI = −23+/−6, n = 3); GDF7 (CI = 10+/−10, n = 3). B: Dose response curves for chemotaxis of WEHI 274.1 cells in response to BMP7, BMP6 and GDF7 (n≥3 for each protein at each concentration). Migration in the presence of BMP7 differs significantly from migration in the presence of BMP6 or of GDF7; (*) p<0.05; (**) p<0.01; (***) p<0.002 (Student's t test).

To study the mechanisms underlying the rapid, chemotropic effects of BMPs, we tested the chemotactic responses of two monocytic cell lines, mouse WEHI 274.1 cells and human THP-1 cells. Transwell chemotaxis assays were used to measure the migration of cells from the upper to the lower chamber, across a 5 µm pore filter, in response to candidate chemotactic agents. The response was measured as the number of cells that migrated into the transwell filter pores normalized to the number of cells in the pores under control conditions, providing a chemotactic index (CI). BMP7 in the lower chamber evoked migration of WEHI 274.1 and THP-1 cells (Figures 1A and S1, respectively). The responses to 10 pg/ml BMP7 (WEHI 274.1: CI = 93+/−38, Figure 1A; THP-1: CI = 125+/−31, Figure S1) were comparable to the response to the standard chemotactic agent, Monocyte Chemotactic Protein-1 (MCP-1) at 100 ng/ml (WEHI 274.1: CI = 139+/−45, Figure 1A; THP-1: CI = 71+/−18, Figure S1). BMP7 showed a biphasic activity profile, with peak chemotactic activity at 1 pg/ml (CI = 236+/−21; Figure 1B). The effects of neutralizing and reversing the BMP7 gradient at all concentrations tested confirmed that the response represents directed migration, or chemotaxis, rather than activation of non-directional chemokinetic activity (Table S1).

### A Subset of BMPs Exhibit Chemotactic Activity

To gain insight into the ligand/receptor relationships of chemotropic BMPs, we examined the profile of BMPs that evoke chemotaxis. We tested a range of BMPs belonging to different subgroups based on structural similarity [30], [31]. BMP6, a member of the BMP5/6/7 structural subgroup, BMP2 and BMP4, from the BMP2/4 subgroup, and GDF7, representing the GDF5/6/7 subgroup, were all tested, initially at 10 pg/ml. BMP2 stimulated chemotaxis in WEHI 274.1 cells (CI = 116+/−40; Figure 1A) and THP-1 cells (CI = 89+/−21; Figure S1), as did BMP4 in WEHI 274.1 cells (J. C. Perron and J. Dodd, unpublished). In contrast, BMP6 (WEHI 274.1: CI = −23+/−6; THP-1: CI = 4+/−17) and GDF7 (WEHI 274.1: CI = 10+/−10; THP-1: CI = 6+/−10,) were unable to stimulate monocytic cell chemotaxis (Figures 1A and S1). The similarity of the pattern of responses of the mouse and human monocytic cells to BMPs, and the close resemblance of the BMP7 responses in the two cell lines to those observed with primary monocytes [7], led us to focus on only one of the lines, WEHI 274.1, for all further experiments described below.

To assess whether the inability of BMP6 and GDF7 to stimulate chemotaxis resulted from differences in potency, dose response curves were performed using BMP6, GDF7, BMP7, BMP4 and BMP2 over a wide range of concentrations (10 fg/ml–100 ng/ml [0.3 fM–0.3 nM]). BMP2 and BMP4 both mimicked BMP7, showing a biphasic dose response curve (J. C. Perron and J. Dodd, unpublished). In contrast, neither BMP6 nor GDF7 stimulated WEHI 274.1 cell chemotaxis at any concentration tested (Figure 1B).

Both BMP6 and GDF7 are known to have inductive activity in neural tissue *in vitro* [32]. Therefore, in control experiments, to confirm that these BMPs were active, we tested for BMP-induced expression of the LIM HD transcription factor, LH2, in explants of chick neural tube, as previously described [33]. BMP6 and GDF7 both induced robust expression of LH2 that was indistinguishable from that induced by BMP7 (Figure S2A). We then tested whether BMP6, BMP7 and GDF7 activate the classical signaling pathway in WEHI 274.1 cells, by measuring the phosphorylation of R-Smads by Western blot analysis of BMP-treated WEHI 274.1 cell extracts. All three BMPs promoted R-Smad phosphorylation, to similar levels, in WEHI 274.1 cells (Figure S2B). Thus, although BMP6, BMP7 and GDF7 share the ability to stimulate early classical transduction events downstream of canonical BMP receptor binding, BMP6 and GDF7 are unable to activate the chemotactic response.

Together, these initial experiments in WEHI 274.1 and THP-1 cells demonstrate a BMP-evoked chemotropic activity that exhibits agonist selectivity. The profile of chemotactic activity among BMPs suggests that the composition of the receptor complexes and downstream signaling pathways underlying the chemotactic response are distinct from those mediating transcriptional responses to BMPs. Nonetheless, it remains unclear whether these pathways comprise non-classical transduction components or include classical components, commandeered to elicit chemotropic responses.

### Canonical Type II BMP Receptors Are Required for the Chemotactic Response of WEHI 274.1 Cells to BMP7

**Figure 2:**
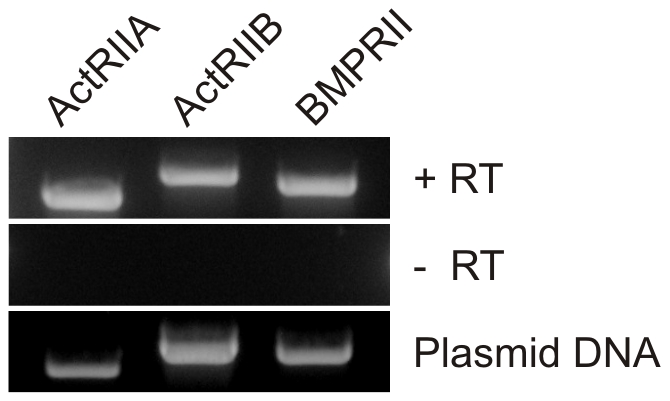
Type II BMP receptor mRNA expression in WEHI 274.1 cells. cDNA from WEHI 274.1 cells transcribed from total RNA in the presence (top, +RT) and absence (middle, -RT) of reverse transcriptase and amplified with BMP receptor subunit-specific primers. Plasmids encoding type II BMP receptor cDNAs (bottom, Plasmid DNA) were used as a positive control for each primer set. The sizes of the expected reaction products are 702 bp for ActRIIA, 928 bp for ActRIIB, and 813 bp for BMPRII.

To determine whether canonical BMP receptors mediate chemotropic responses to BMPs, and in light of the noted association of type II BMP receptors with cytoskeletal signaling elements, we focused on the roles of type II BMP receptors in BMP7-evoked chemotaxis. We first determined which type II BMP receptor subunits are expressed by WEHI 274.1 cells, using RT-PCR analysis to detect BMP receptor mRNA. All three known type II BMP receptor subunits, ActRIIA, ActRIIB and BMPRII, were found to be expressed in WEHI 274.1 cells (Figure 2).

**Figure 3:**
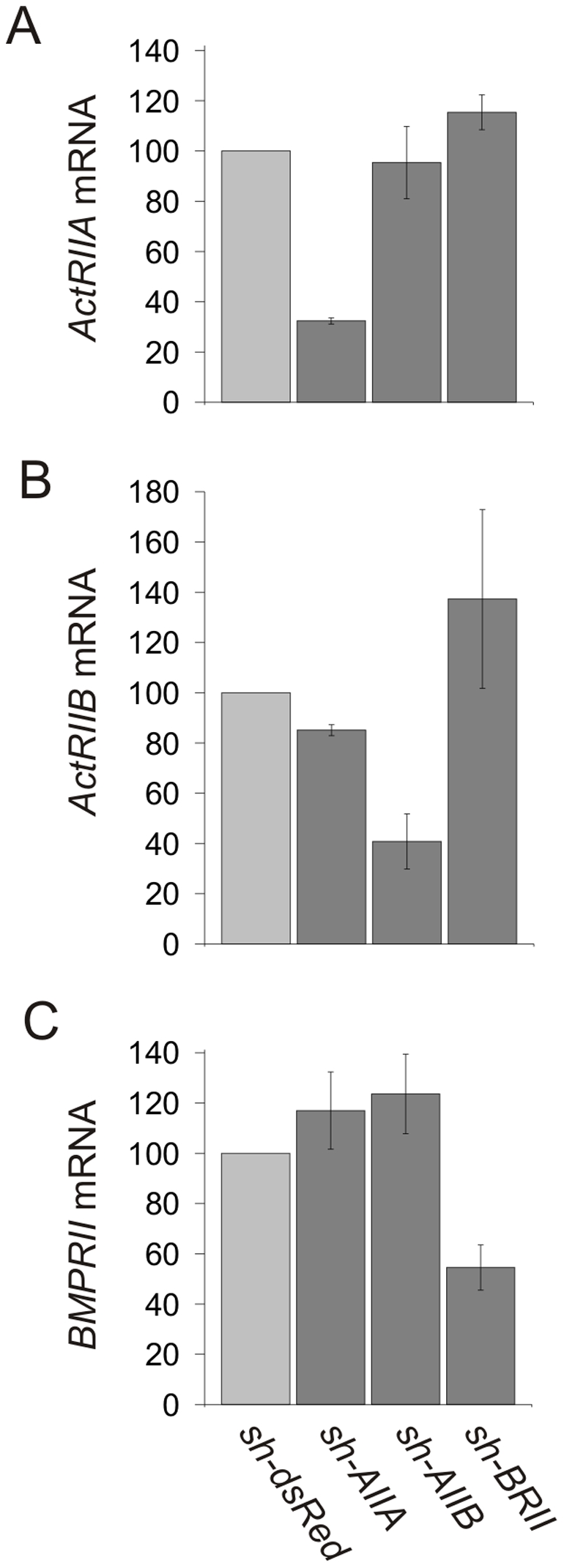
Knockdown of type II BMP receptors in WEHI 274.1 cells by receptor-targeted shRNAs. Real-time QPCR analysis of dsRed^Δ^−, ActRIIA^Δ^−, ActRIIB^Δ^− and BMPRII^Δ^WEHI cells. Results are expressed as the percent of control (mean +/− SEM) for each condition relative to BMP receptor mRNA levels in dsRed^Δ^WEHI (control) cells (n = 2). A: *ActRIIA* mRNA expression in the presence of *sh-AIIA* (32%+/−1%), *sh-AIIB* (95%+/−14%) or *sh-BRII* (115%+/−7%). B: *ActRIIB* mRNA expression in the presence of *sh-AIIA* (85%+/−2%), *sh-AIIB* (41%+/−11%) or *sh-BRII* (137%+/−36%). C: *BMPRII* mRNA expression in the presence of *sh-AIIA* (117%+/−15%), *sh-AIIB* (124%+/−16%) or *sh-BRII* (55%+/−9%). Individual type II BMP receptor shRNAs modulated target mRNAs selectively, but had no significant effect on the expression level of non-target BMP receptor mRNA.

To examine the requirement for type II BMP receptors in BMP7-evoked WEHI 274.1 cell chemotaxis, we tested the effect of blocking expression of individual receptor subunits by RNAi. To do so, we developed short hairpin RNA (shRNA) vectors targeting mouse *ActRIIA*-, *ActRIIB*- and *BMPRII*-specific sequences and also encoding for GFP to label shRNA-expressing cells. BMP receptor shRNAs were electroporated individually into WEHI 274.1 cells (^Δ^WEHI), which were sorted to enrich for the GFP-expressing population. As a negative control, WEHI 274.1 cells were electroporated with a shRNA that targets mRNA encoding the red fluorescent protein, dsRed (*sh-dsRed*). Quantitative PCR (QPCR) was used to measure the effects of expressing subunit-specific shRNAs on endogenous levels of type II receptor mRNA in WEHI 274.1 cells (Figure 3). Compared with BMP receptor mRNA levels in dsRed^Δ^WEHI cells, each type II BMP receptor shRNA significantly inhibited expression of its target receptor in sorted WEHI 274.1 cells: *sh-AIIA* (70% reduction of ActRIIA mRNA; Figure 3A), *sh-AIIB* (60% reduction of ActRIIB mRNA; Figure 3B) and *sh-BRII* (46% reduction of BMPRII mRNA; Figure 3C). Expression of individual BMP receptor shRNAs had no significant effect on the relative levels of non-target type II BMP receptor mRNA expression (Figure 3).

It was not possible to measure commensurate regulation of endogenous receptor subunit protein in WEHI 274.1 cells because antibodies that are selective for individual type II BMP receptor subunits detected protein only at levels achieved by transient over expression. The effect of each shRNA construct on type II BMP receptor subunit protein expression was therefore tested by co-transfection with target and non-target receptor cDNAs in HEK 293 cells. Receptor subunit expression was measured on Western blots of transfected cell lysates. Heterologous expression of BMP receptor protein was unaffected by co-expression with *sh-dsRed* compared with empty vector (pLL3.7) controls (Figure S3). In contrast, co-transfection of *sh-AIIA*, *sh-AIIB* and *sh-BRII* reduced target protein expression effectively (Figure S4A, S4E, S4I, respectively). Moreover, as was shown for mRNA in WEHI 274.1 cells, each shRNA was target specific and did not cause a reduction in the expression of non-target BMP receptor proteins (Figure S4). Thus, shRNA expression in WEHI 274.1 or HEK 293 cells effectively and selectively reduced expression of each of the three type II BMP receptor subunits.

**Figure 4:**
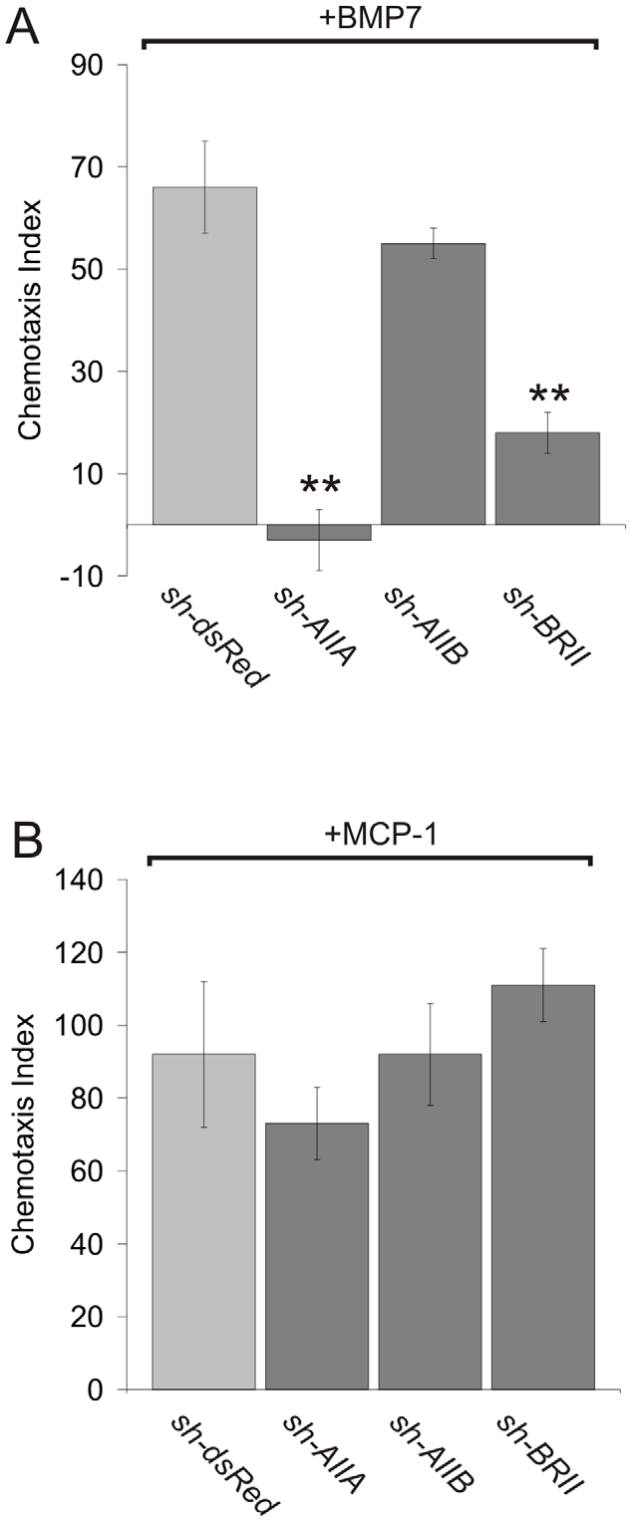
BMP7-stimulated WEHI 274.1 cell chemotaxis following type II BMP receptor knockdown. Chemotaxis of dsRed^Δ^−, ActRIIA^Δ^−, ActRIIB^Δ^− and BMPRII^Δ^WEHI cells (mean +/− SEM) in response to BMP7 (10 pg/ml, A) or MCP-1 (100 ng/ml, B). In A, dsRed^Δ^WEHI (CI = 66+/−9; n = 8), ActRIIA^Δ^WEHI (CI = −3+/−6; n = 3), ActRIIB^Δ^WEHI (CI = 55+/−3; n = 4) and BMPRII^Δ^WEHI cells (CI = 18+/−4; n = 7). n = 3 for each condition in B. BMP7-stimulated chemotaxis following knockdown with either *sh-AIIA* or *sh-BRII* differs significantly from that in dsRed^Δ^WEHI (control) cells; (**) p<0.0001 (Student's t test).

We then asked whether the reduction of expression of individual receptor subunits changes the response of WEHI 274.1 cells to BMP7. WEHI 274.1 cells expressing either type II BMP receptor shRNAs or *sh-dsRed* were tested in chemotaxis and Smad phosphorylation assays. We found that *sh-AIIA* eliminated the chemotactic response to BMP7 (CI = −3+/−6; Figure 4A) compared to the response in dsRed^Δ^WEHI cells (CI = 66+/−9; Figure 4A). *sh-BRII* also effectively inhibited BMP7-evoked chemotaxis (73% reduction; CI = 18+/−4; Figure 4A). Expression of *sh-AIIB*, however, had no effect on the response of WEHI 274.1 cells to BMP7 (17% reduction; CI = 55+/−3; Figure 4A). In control experiments, using MCP-1 as an agonist, ActRIIA^Δ^−, ActRIIB^Δ^− and BMPRII^Δ^WEHI cells all showed normal chemotactic responses (Figure 4B).

To determine whether other BMP responses were affected by loss of individual type II receptors, in particular, ActRIIA or BMPRII, we examined the ability of BMP7 to stimulate phosphorylation of R-Smads in ActRIIA^Δ^−, ActRIIB^Δ^− and BMPRII^Δ^WEHI cells. Phosphorylation was unchanged in all three knockdown conditions compared to control, dsRed^Δ^WEHI cells (Figure S5). Together, these results provide evidence that the canonical type II BMP receptor subunits, ActRIIA and BMPRII, are both required for BMP7-evoked chemotaxis but that ActRIIB is not required. In contrast, this requirement and selectivity for the function of individual receptors is not reflected in the activation of the classical Smad-dependent pathway in response to BMP7, where receptor activity may be redundant [34].

### Actriib Cannot Rescue BMP7-Evoked Chemotaxis Following Knockdown of Actriia Expression

Although the results of the RNAi experiments revealed a selective requirement in BMP7-evoked chemotaxis for ActRIIA and BMPRII receptor subunits, the question remained whether ActRIIB has the ability to mediate the chemotactic response to BMP7. We therefore tested whether exogenously expressed mouse ActRIIB is sufficient to compensate for the loss of ActRIIA in WEHI 274.1 cells. The selectivity of each shRNA reagent for its target mRNA, endogenously expressed in WEHI 274.1 cells or heterologously expressed in transfected in HEK 293 cells (see Figures 3 and S4), made such a rescue assay feasible.

**Figure 5:**
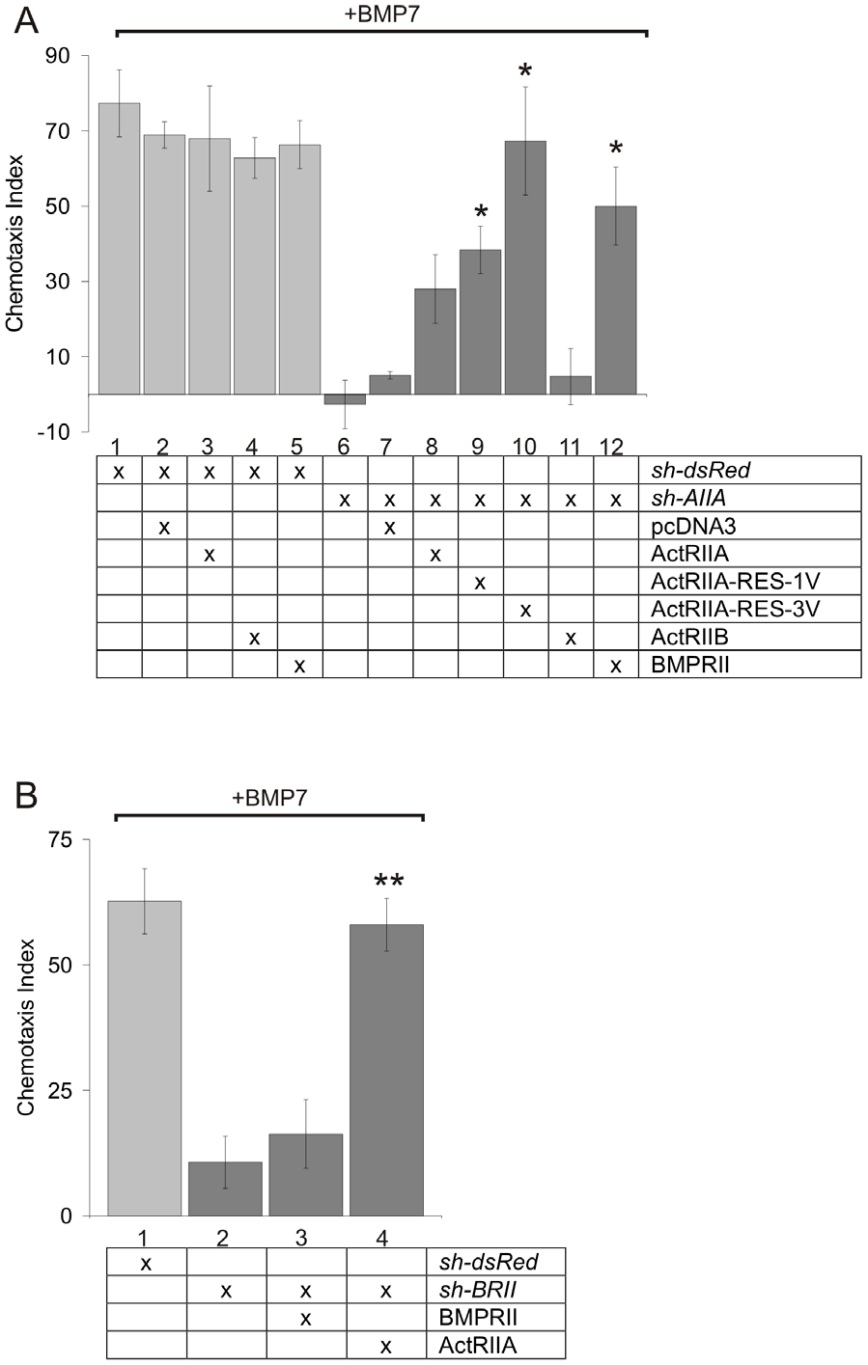
Rescue of shRNA-mediated inhibition of BMP7-stimulated chemotaxis by ActRIIA and BMPRII but not ActRIIB. A: Chemotaxis in response to 10 pg/ml BMP7 (mean +/− SEM) of dsRed^Δ^WEHI cells (Lanes 1–5): alone (CI = 77+/−9, n = 3) or co-expressing pcDNA3 (CI = 69+/−4, n = 2), ActRIIA (CI = 68+/−14, n = 3), ActRIIB (CI = 63+/−5, n = 2) or BMPRII (CI = 66+/−6, n = 2). These are compared with ActRIIA^Δ^WEHI cells (Lane 6–12): alone (CI = −3+/−6, n = 3) or co-expressing pcDNA3 (CI = 5+/−1, n = 3), wild-type ActRIIA (CI = 28+/−9, n = 3), ActRIIA-RES-1V (CI = 38+/−6, n = 3), ActRIIA-RES-3V (CI = 67+/−14, n = 3), ActRIIB (CI = 5+/−8, n = 3) or BMPRII (CI = 50+/−10, n = 3). B: Chemotaxis in response to 10 pg/ml BMP7 (mean +/− SEM) of dsRed^Δ^WEHI (CI = 63+/−7) and BMPRII^Δ^WEHI (CI = 11+/−5) cells (Lanes 1 and 2) compared with BMPRII^Δ^WEHI cells co-expressing wild-type BMPRII (CI = 16+/−7) or ActRIIA (CI = 58+/−5) (Lanes 3 and 4). n = 3 for each condition. BMP7-evoked chemotaxis in ActRIIA^Δ^WEHI cells expressing ActRIIA-RES-1V, ActRIIA-RES-3V or BMPRII differs significantly from chemotaxis in ActRIIA^Δ^WEHI cells alone; (*) p<0.02 (Student's t test). BMP7-evoked chemotaxis in BMPRII^Δ^WEHI cells expressing wild-type ActRIIA differs significantly from chemotaxis in BMPRII^Δ^WEHI cells alone; (**) p<0.005 (Student's t test).

To develop the assay, we first demonstrated that over expression of ActRIIA, ActRIIB or BMPRII in dsRed^Δ^WEHI cells did not affect the baseline chemotactic response of the cells to BMP7 (Figure 5A, lanes 3–5). Next, we tested the ability of wild-type mouse ActRIIA and *sh-AIIA*-resistant ActRIIA mutant cDNAs to rescue BMP7-stimulated chemotaxis in ActRIIA^Δ^WEHI cells. Expression levels of ActRIIA protein generated by wild-type and shRNA-resistant cDNAs were measured in parallel by Western blot analysis of COS-1 cell lysates co-transfected with *sh-AIIA*. As shown earlier in HEK 293 cells (see Figure S4A), ActRIIA protein was almost undetectable in COS-1 cells co-expressing wild-type ActRIIA and *sh-AIIA* (Figure S6), confirming susceptibility to the shRNA. In contrast, co-transfection of *sh-AIIA*-resistant ActRIIA cDNAs (ActRIIA-RES-1V and ActRIIA-RES-3V) with *sh-AIIA* permitted ActRIIA protein expression at levels similar to that in *sh-dsRed*-transfected cells (Figure S6). In chemotaxis assays, co-expression of control vector (pcDNA3) had no effect on the normal response to BMP7 in dsRed^Δ^WEHI cells (Figure 5A, lanes 1 and 2) or the loss of chemotaxis observed in ActRIIA^Δ^WEHI cells (Figure 5A, lanes 6 and 7). In contrast, co-expression of exogenous wild-type mouse ActRIIA with *sh-AIIA* in WEHI 274.1 cells resulted in a minor recovery of chemotaxis compared to that in control cells (∼40% recovery of CI; Figure 5A, lane 8). Moreover, co-expression of *sh-AIIA*-resistant ActRIIA cDNA mutants restored BMP7-evoked chemotaxis, despite the presence of the potent *sh-AIIA* shRNA. ActRIIA^Δ^WEHI cells co-expressing ActRIIA-RES-1V showed an intermediate response (∼50% recovery of CI) and co-expression of ActRIIA-RES-3V, resulted in almost full rescue (∼90% recovery of CI) of BMP7-evoked chemotaxis (Figure 5A, lanes 9 and 10, respectively). Thus, restoration of ActRIIA expression was sufficient to overcome *sh-AIIA*-mediated inhibition of the chemotactic response to BMP7.

We then tested whether over expression of ActRIIB protein is able to rescue the chemotactic response to BMP7 in WEHI 274.1 cells in which endogenous ActRIIA has been selectively silenced. Transient transfection in HEK 293 cells resulted in strong expression of ActRIIB receptor protein which was not affected by co-transfection of *sh-AIIA* (see Figure S4D). However, expression of exogenous ActRIIB in ActRIIA^Δ^WEHI cells did not rescue the chemotactic response to BMP7 (Figure 5A, lane 11), providing strong support for the idea that ActRIIB cannot, alone, or in combination with endogenous BMPRII, mediate BMP7-dependent chemotaxis.

Finally, we used the rescue assay to explore whether the selective requirement for endogenous ActRIIA and BMPRII subunits in BMP7-evoked chemotaxis is absolute. Expression of exogenous BMPRII restored BMP7-evoked chemotaxis in ActRIIA^Δ^WEHI cells to ∼70% of control activity (Figure 5A, lane 12), indicating that when over expressed BMPRII counters the effect of loss of ActRIIA. In reciprocal experiments, although expression of wild-type, *sh-BRII-*sensitive, BMPRII failed to restore BMP7-stimulated chemotaxis in BMPRII^Δ^WEHI cells (Figure 5B, lane 3), introduction of exogenous, wild-type ActRIIA fully rescued the response to BMP7 (∼90% recovery of CI; Figure 5B, lane 4). Thus, although ActRIIA and BMPRII are both essential mediators of the chemotactic response of WEHI 274.1 cells to BMP7, at the non-physiological concentration achieved by transient over expression either receptor is capable of sustaining the response alone. In contrast, ActRIIB is not required for the chemotactic response and cannot support this function.

### BMP7-Evoked Chemotaxis Does Not Require Smad-Dependent Signaling

Our results suggest that the chemotropic response of WEHI 274.1 cells to BMP7 depends on the selective engagement of ActRIIA and BMPRII receptor subunits and raise the possibility that engagement of this particular receptor complex results in coupling selectively to an intracellular signaling pathway that drives chemotaxis. All BMPs tested in this study stimulated R-Smad phosphorylation, to similar levels, in WEHI 274.1 cells (see Figure S2B), suggesting the differential ability of some BMPs to stimulate chemotaxis is not imparted by selective activation of R-Smads. However, our experiments did not distinguish between R-Smad isoforms. Therefore to examine the role of the classical Smad cascade further we asked whether the co-Smad, Smad4, a key mediator of BMP-dependent gene transcription and downstream of R-Smad activation, is required for BMP7-evoked chemotaxis.

**Figure 6:**
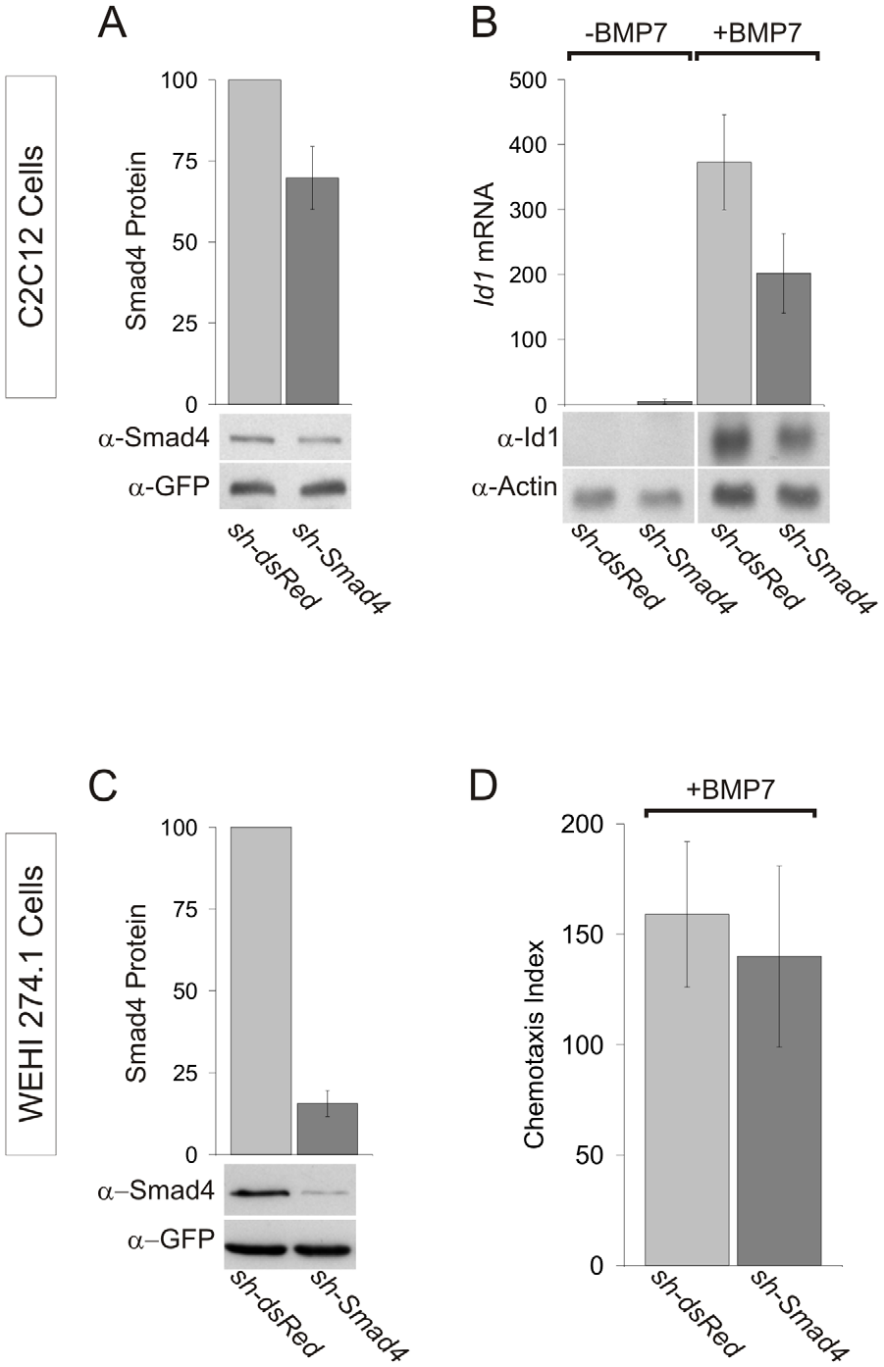
Knockdown of Smad4 inhibits BMP7-evoked *Id1* induction but not BMP7-evoked chemotaxis. A: Western blot analysis of lysates of dsRed^Δ^C2C12 and Smad4^Δ^C2C12 cells, probed for expression of Smad4 and GFP (loading control) and showing 30% knockdown of endogenous Smad4 (mean +/− SEM, n = 3). B: Northern analysis of mouse *Id1* expression in control and 150 ng/ml BMP7-stimulated dsRed^Δ^C2C12 and Smad4^Δ^C2C12 cells (*Actin* expression used as loading control). Smad4^Δ^C2C12 cells show a 46% decrease in BMP7-evoked *Id1* induction (mean +/− SEM, n = 4). C: Western blots of lysates from dsRed^Δ^WEHI and Smad4^Δ^WEHI cells, probed for expression of Smad4 and GFP (loading control), demonstrate efficient knockdown of endogenous Smad4 by *sh-Smad4* (84% reduction, mean +/− SEM, n = 3) in WEHI 274.1 cells. D: dsRed^Δ^WEHI and Smad4^Δ^WEHI cell chemotaxis in response to 10 pg/ml BMP7 did not differ (mean +/− SEM, n = 3). dsRed^Δ^WEHI (CI = 159+/−33) v. Smad4^Δ^WEHI (CI = 140+/−41); p = 0.745 (Student's t test).

A shRNA construct targeting *Smad4* RNA (*sh-Smad4*) was generated and tested for the ability to reduce expression and activity of Smad4 in the classical BMP pathway. To test whether the *sh-Smad4* construct efficiently silences Smad4 function, we used a common assay of BMP-evoked induction of the early response gene *Id1* in C2C12 myoblasts [35], [36]. In C2C12 cells transfected with *sh-Smad4* (approximately 55% transfection efficiency measured by GFP expression), Smad4 protein levels decreased by 30% (Figure 6A) and BMP7-stimulated induction of *Id1* mRNA expression was reduced by 46% (Figure 6B), demonstrating a concurrent decrease in Smad4 expression and activity following *sh-Smad4* expression. We next tested the effects of *sh-Smad4* in WEHI 274.1 cells, sorted to enrich for GFP-expressing cells. Smad4 protein expression was reduced in Smad4^Δ^WEHI cells by 84% relative to the dsRed^Δ^WEHI cell population (Figure 6C). However, inhibition of Smad4 activity in WEHI 274.1 cells had no effect on BMP7-stimulated chemotaxis (Figure 6D), suggesting an alternate pathway underlies activation of cytoskeletal elements mediating BMP7 stimulation of chemotaxis.

### BMP7 Requires Activation of a Non-Canonical Signaling Pathway

Divergence from the inductive pathway may reside in direct coupling of an ActRIIA:BMPRII-containing receptor complex to a cascade of cytoskeletal regulators or occur downstream of R-Smad phosphorylation. The association of PI3K with BMP and TGFβ receptor subunits [24], [37] and the dependence on PI3K activity for signaling by chemotactic factors other than BMPs [27], [28] led us to explore the possibility that BMP7 stimulates PI3K activity and whether PI3K-dependent activity is required for BMP7-evoked chemotropic responses.

**Figure 7:**
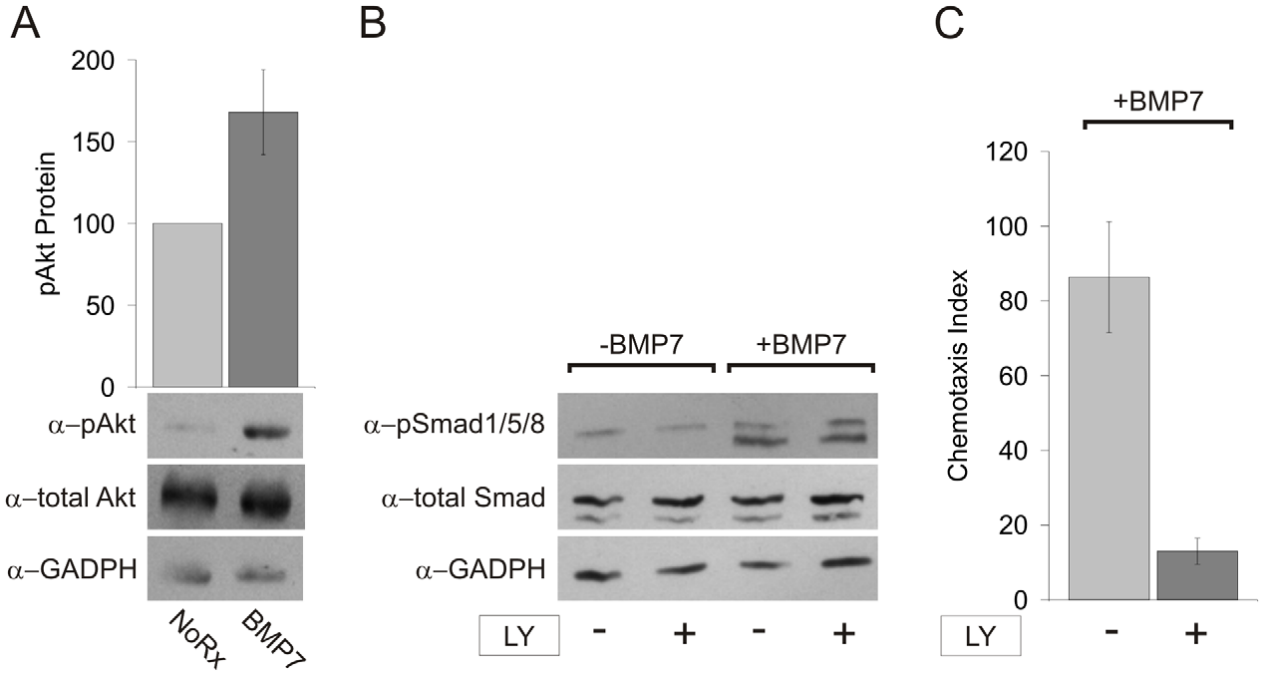
PI3K activity is stimulated by BMP7 and required for BMP7-evoked chemotaxis. A: Western blots of whole cell lysates of WEHI 274.1 cells incubated with or without 50 ng/ml BMP7 were probed with a phospho-specific α-Akt(S) antibody. Measurement of total Akt and GAPDH provided loading controls. Densitometric measurement (mean+/−SEM) shows an increase in response to BMP7 (70% over control n = 2). B: Western blots of whole cell lysates of control and 50 µM LY-treated WEHI 274.1 cells incubated with or without 50 ng/ml BMP7 were probed with a phospho-specific α-Smad1/5/8 antibody. Measurement of total Smad and GAPDH provided loading controls. Inhibition of PI3K activity did not affect BMP7-evoked stimulation of R-Smad phosphorylation. C: Chemotaxis (mean +/− SEM) of WEHI 274.1 cells in response to 10 pg/ml BMP7 was significantly reduced following 50 µM LY294002 (LY) treatment (85% reduction). BMP7-LY (CI = 86+/−15, n = 3) v. BMP7+LY (CI = 13+/−3.5, n = 3), p = 0.0086 (Student's t test). Treatment with LY alone had no effect on WEHI 274.1 cell movement under control conditions (LY alone: CI = 3.5+/−2.5, n = 2; J. C. Perron and J. Dodd, unpublished).

The phosphorylation of Akt is a commonly used indicator of PI3K activation [18]. We therefore measured Akt phosphorylation on S473 in WEHI 274.1 cells and found that BMP7 stimulated a robust phosphorylation (Figure 7A). To examine the dependence of BMP7-evoked chemotaxis on PI3K activity, WEHI 274.1 cells were pre-incubated with the PI3K inhibitors, LY294002 (LY) or Wortmannin (WM), and then tested for their ability to respond to BMP7. To control for non-specific effects of LY or WM, the ability of BMP7 to stimulate phosphorylation of R-Smads in the presence of the inhibitors was also tested. BMP7-evoked phosphorylation of R-Smads by was unaffected by LY or WM treatment (Figures 7B and S7A, respectively). In contrast, in chemotaxis assays, both LY and WM treatment inhibited BMP7-stimulated chemotaxis (LY: 85% reduction, Figure 7C and WM: 76% reduction, Figure S7B). Taken together, these data demonstrate that stimulation of chemotaxis by BMP7 involves a signaling pathway, independent of Smad activation, which influences cytoskeletal reorganization through activation of PI3K-dependent mechanisms.

## Discussion

BMPs exert short latency, tropic effects on local cytoskeletal dynamics, causing reorientation of cells or parts of cells, such as growth cones, as well as classical, long-term trophic effects stimulating growth and differentiation. Such activities can occur in the same cell, raising the question of how the distinct responses to BMPs are transduced and whether components of the classical pathway support both types of response. Here, we demonstrate BMP agonist selectivity for chemotactic activity and show that the type II BMP receptor subunits ActRIIA and BMPRII mediate BMP7-evoked chemotaxis. This specific type II receptor combination does not appear to be required for BMP7-mediated activation of the Smad cascade and resulting inductive activity. Our evidence suggests that the events downstream of ActRIIA and BMPRII receptor subunit activation, leading to chemotaxis, do not rely on Smad4-dependent signaling but instead involve signaling through a PI3K-dependent pathway. The concordance between the identity of BMPs that can stimulate monocytic cell chemotaxis and those that have reorienting activity in other cell types suggests that the mechanism of engagement of a selective type II receptor subunit pair in the receptor complex, leading to activation of a Smad-independent intracellular pathway, may have significance for the chemotropic activities of BMPs in a wide range of cell types.

### BMPs as Chemotropic Agents: Agonist Selectivity

A general issue in understanding the mechanisms underlying the great variety of BMP inductive and tropic activities in the developing and mature organism is that of how a large number of BMPs act through a restricted number of BMP receptors to give rise to many different outcomes. Selectivity may be provided by diverse forms of agonist-receptor interaction that generate differential downstream targeting [38]–[41]. The chemotropic versus inductive effects of BMPs represent extreme examples in the spectrum of this problem and knowledge of agonist selectivity and transduction mechanisms are likely to illuminate the general issue.

The BMPs examined in this study all have trophic activity demonstrable by neural induction ability (see Figure S2A) [11], [32], [33], [42], by phosphorylation of R-Smads (see Figure S2B) and by Smad4-dependent induction of *Id1* (see Figure 6). They presumably activate these transcriptional events through classical pathways involving canonical BMP receptors. However, our results show that it is not possible to predict chemotropic activity of BMPs solely on the basis of their inductive activities. The agonist selectivity that we observed is also surprising in the light of structural considerations: BMP7, BMP2 and BMP4 were all active in promoting chemotaxis yet belong to distinct structural subgroups [30], [31]. In contrast, BMP7 and BMP6 belong to the same structural BMP subgroup (BMP5/6/7) and are 87% identical, yet BMP6 was inactive in monocytic cell chemotaxis. Nonetheless, the same activity profile has been observed for reorientation of the growth cones of embryonic dorsal spinal neurons ([11], [12] and J. C. Perron and J. Dodd, unpublished), providing support for the notion of a group of chemotropic BMPs, defined not by structural similarity but by ability to activate a selective intracellular pathway leading to cytoskeletal reorganization, and suggesting common underlying mechanisms.

**Figure 8:**
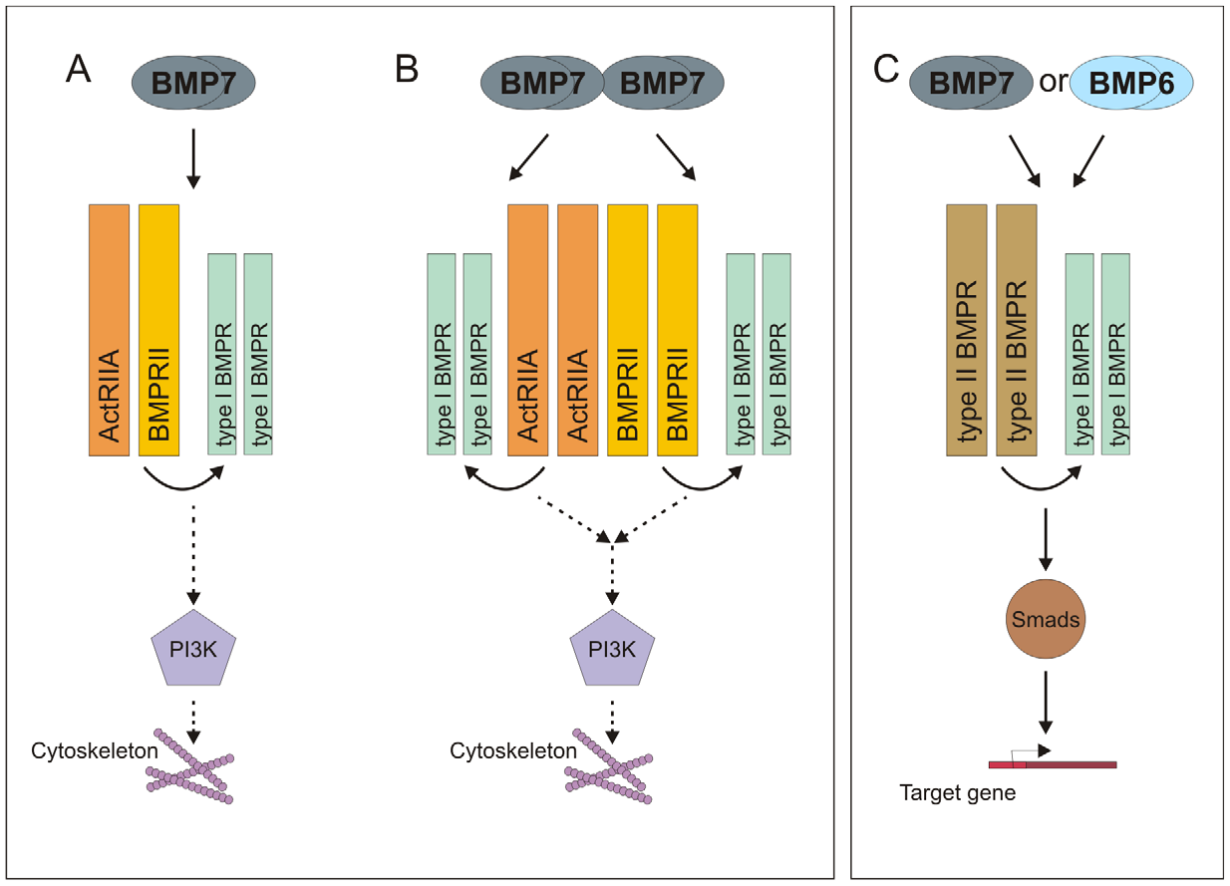
Model of differential receptor subunit recruitment and signaling underlying BMP-evoked chemotaxis and trophic actions. We have demonstrated that ActRIIA and BMPRII receptor subunits are both selectively required for BMP7-evoked chemotaxis in monocytic cells. How do the individual receptor subunits contribute to this activity? Do they work together in a common complex? A,B: Our data support a model in which BMP7 engages both ActRIIA and BMPRII subunits either as part of a heteromeric type II receptor pair (A) or as homodimers, possibly forming unique receptor complexes combining pairs of each of the type II receptor subunits (B). The identity of the type I receptors that contribute to this complex is not known, although recent evidence implicates BMPRIB in BMP-dependent axon guidance [48], [49]. Engagement of ActRIIA and BMPRII subunits by chemotropic BMPs activates a signaling pathway leading to PI3K activity and cytoskeletal reorganization that is independent of Smad4-mediated nuclear signaling. C: In contrast, BMP6 does not stimulate chemotaxis, presumably being unable to recruit or activate the ActRIIA:BMPRII complex necessary for chemotropic signaling. In the same cell, however, BMP7 and BMP6 share the ability to activate Smad-dependent transcriptional pathways, through receptor mechanisms that do not depend selectively on either ActRIIA or BMPRII.

Do chemotropic BMPs, typified by BMP7, and non-chemotropic BMPs, such as BMP6, display different receptor binding capacities? Studies in heterologous expression systems have shown that BMP7 and BMP6 have the capacity to bind to the same type I and type II BMP receptor subunits [19], although subtleties of binding may be masked by the high concentrations achieved by over expression, potentially concealing receptor specificity that exists in a cell-specific context. For example, differences in affinity or structure at receptor interfaces may account for differential binding and, consequently, differential signaling by BMPs [41]. Nonetheless, selective binding of individual receptor subunits may not alone explain the differential activities of closely related BMPs. Specificity may lie rather in the recruitment of particular combinations of subunits, such that chemotropic BMPs engage a receptor complex whose composition is distinct from complexes mediating inductive responses evoked by a majority of BMPs (Figure 8). Alternatively, chemotropic BMPs may activate a common receptor complex in a novel way, perhaps by also engaging accessory proteins [43], [44], that selectively transduces information to the cytoskeleton.

### Coordinated Type II BMP Receptor Subunit Activity Mediates Acute Effects of BMP7

Our findings provide insight into the composition of the type II BMP receptor subunit pair activated in the chemotropic response to BMPs. The efficient knockdown of individual receptors in WEHI 274.1 cells (70% *ActRIIA*, 60% *ActRIIB*, 45% *BMPRII*) using RNAi provided a means to test the relative contributions of the individual type II BMP receptor subunits. We show that all three known type II BMP receptors are expressed by WEHI 274.1 cells but that only ActRIIA and BMPRII are required for function. Knockdown of *ActRIIB* mRNA in WEHI 274.1 cells has no effect on the chemotactic response to BMP7. Moreover, ActRIIB cannot mediate a chemotactic response when over expressed in cells in which ActRIIA is reduced. The demonstration that both ActRIIA and BMPRII receptor subunits are selectively and individually required for chemotactic activity argues against the idea that wild-type levels of the remaining potential receptor subunit pairs, homodimers of either ActRIIA or BMPRII, homodimers of ActRIIB or heterodimers of ActRIIB with either ActRIIA or BMPRII, independently mediate the chemotactic response. Together, our findings therefore fit best with a scheme in which chemotropic BMPs, typified by BMP7, engage a receptor complex in which the type II component is a heterodimer of ActRIIA and BMPRII (Figure 8A). Nevertheless, the incomplete knockdown of ActRIIB achieved in our experiments leaves open the possibility that this receptor subtype contributes in some way to the chemotactic response, albeit at a different threshold than that of ActRIIA and BMPRII.

The notion of selective subunit pairing to produce different signaling outcomes extends the finding that recruitment of different combinations of type I and type II subunit homodimers that couple to distinct downstream signaling pathways underlies some of the differential activities of the TGFβ superfamily [38], [45]. However, previous work in many systems has emphasized the roles of homomeric pairs of type I and type II receptor subunits in BMP signaling and, for a variety of technical reasons, the potential role of heteromeric pairings has not been addressed. Studies in which the specific association of a type I BMP receptor with existing clusters of BMPRII is necessary for the initiation of efficient Smad signaling [39], [40], emphasize the importance of understanding the mode of receptor complex oligomerization in directing signaling outcomes for distinct ligands and receptors. The heterodimeric model above (Figure 8A) assumes that, like other BMP receptor complexes [19], [20], only two type II subunits contribute to the complex. However, the results of the rescue experiments described here (see Figure 5) indicate that, at high enough concentration, homodimers of either ActRIIA or BMPRII, but not ActRIIB, can support BMP7-dependent chemotaxis. Although the non-physiological levels of receptor subunits produced by over expression might only artificially allow type II BMP receptor homodimers to mediate the chemotactic response to BMP7, this finding raises the possibility that a threshold level of cell surface receptor expression, comprising either ActRIIA or BMPRII homodimers, is sufficient for a chemotactic response to BMPs. As described above, we show that in the context of either ActRIIA or BMPRII knockdown, the unaffected, endogenous type II BMP receptor is unable to compensate for the loss of the other. Nonetheless, the levels of activity achieved by the coordinate activation of endogenous ActRIIA and BMPRII homodimers in wild-type conditions may be sufficient to elicit a chemotactic response. Such a model (Figure 8B) would require the formation of a receptor complex comprising multiple pairs of subunits and appropriate BMP dimer orientation. This notion of a multi-unit complex represents a less parsimonious model than that of a complex in which the type II receptor pair consists of a heterodimeric ActRIIA:BMPRII unit (Figure 8A). However, neither model can be dismissed on the current evidence.

The observed selectivity in type II BMP receptor activity in chemotaxis raises two further questions about the formation of receptor complexes. First, how do certain BMPs recruit the ActRIIA and BMPRII receptor subunit pair? Although the selection of downstream signaling pathways has previously been shown to be determined by whether BMPs actively recruit individual receptor subunits sequentially to the ligand/receptor complex or bind to preformed type II/type I receptor pairs [39], [40], it is not clear how this specificity is achieved. Our results do not yet address this issue. Second, how are type I BMP receptor subunits are involved? Type I receptors have generally been associated with Smad-dependent transcriptional signaling and thought to play an instructive role in the induction of cell fate by BMPs [46], [47]. However, two recent studies have implicated type I receptor subunits in axon guidance by BMPs [48], [49] suggesting that recruitment of canonical BMP receptor subunits to the chemotropic pathway may engage both type I and type II receptors. Nonetheless, the demonstration that BMPs bind to preformed pairs of receptors present in the cell membrane [40] leaves open the possibility that type I BMP receptors play a passive role, and that the chemotactic response to BMPs in monocytes is directed by type II BMP receptor pairs.

### Smad4-Independent PI3K Signaling Mediates BMP7-Activated Chemotaxis

How does receptor activation by BMP7 lead to the cellular response culminating in directed cell migration? Is there a dedicated intracellular pathway activated by an ActRIIA:BMPRII receptor complex or does the targeting of the cytoskeleton develop as a branch downstream in the canonical pathway? Several lines of evidence support the idea that BMP7-evoked chemotaxis of WEHI 274.1 cells does not depend on Smad signaling. Like other cells that show chemotropic responses to BMPs, the onset of chemotaxis is too rapid to depend on classical Smad-mediated transcription, translation, maturation and transport of functional protein to the relevant cellular compartment. Moreover, chemotaxis occurs in response to BMPs at concentrations 10–100-fold less than that required for gene induction [7]. Importantly, direct reduction of Smad4 expression and activity has no effect on BMP7-evoked chemotaxis. Finally, R-Smad phosphorylation is robust despite knockdown of individual receptors and loss of chemotaxis. Although we cannot formally exclude a non-transcriptional role for R-Smads in the chemotactic response, BMP6 and GDF7 do not stimulate chemotaxis in WEHI 274.1 cells, yet BMP7, BMP6 and GDF7 all stimulate R-Smad phosphorylation similarly, making it unlikely that the divergence of signaling pathways originates with R-Smad activation. Conversely, we find that BMP7 stimulates PI3K activity and that there is a requirement for PI3K-dependent signaling in BMP7-evoked chemotaxis but not for BMP7-evoked Smad phosphorylation.

These results complement the emerging evidence that members of the TGFβ superfamily, which includes BMPs, regulate cytoskeletal dynamics through Smad-independent intracellular mechanisms. Immunoprecipitation studies have demonstrated an association of the p85 regulatory subunit of PI3K with type II BMP receptors as well as with TGFβ receptors [24], [37] suggesting that type II BMP receptors may directly stimulate PI3K activity and thus play a role in the generation of acute BMP responses. PI3K activity represents a common step in chemotaxis evoked by a number of distinct ligands and it is not yet clear how directly the binding of BMP7 to ActRIIA and BMPRII receptor subunits leads to activation of PI3K. Regardless, Smad4 and PI3K appear to represent components of two divergent signaling paths, one controlling transcriptional activity and the other that gives rise to rapid, directed cell migration. Notably, both PI3K and the small GTPase, Cdc42, appear to be required for the complex but rapid BMP2-mediated stimulation of actin assembly and formation of protrusions during C2C12 myoblast migration [18]. Moreover, TGFβ1 and BMP7 have been shown to stimulate members of the LIMK and Rho GTPase families, influencing cell morphology and actin reorganization, dendritogenesis and growth cone guidance [13], [25], [50], [51]. Several Smad-independent pathways appear therefore to be activated downstream of BMPs in events relying on dynamic regulation of the cytoskeleton.

In summary, the chemotropic effects of BMPs in the WEHI 274.1 cell model system appear to be mediated by canonical BMP receptors combining and responding in non-classical ways to activate Smad4-independent signaling. ActRIIA and BMPRII are required, apparently in a novel heteromeric or multimeric complex, for BMP7-mediated monocytic cell chemotaxis. The ability of BMP7 to evoke Smad-dependent signaling and chemotropic responses in the same cells suggests intricate regulation of these mechanisms. Our findings provide support for the possibility that chemotropic BMPs recruit selectively type II BMP receptor subunits with the capacity to initiate local cytoskeletal signaling.

## Materials and Methods

### Antibodies and Reagents

Recombinant BMPs were purchased from R&D Systems, MCP-1 from Sigma. Antibodies: mouse α-Smad4 (B8), goat α-ActRIIA (N17), rabbit α-ActRIIB (H70) (Santa Cruz), mouse α-BMPRII (BD Transduction Laboratories), rabbit α-phospho-Smad1/5/8, rabbit α-phospho-Akt(S) and rabbit α-Akt (Cell Signaling Technology), rabbit α-Smad1 (Upstate Biotechnology), rabbit α-GFP (Molecular Probes) and rabbit α-GAPDH (Abcam). HRP-conjugated secondary antibodies (HRP goat α-rabbit IgG, HRP goat α-mouse Fcγ, and HRP donkey α-goat IgG) were obtained from Jackson Labs. The receptor antibodies were unable to recognize endogenous receptor protein in WEHI 274.1, HEK 293 or COS-1 cells. Cell culture reagents: DMEM medium, 100x Penicillin/Streptomycin/Glutamine (P/S/G) (Invitrogen) and FBS (Gemini BioProducts). The expression construct for flag-tagged mouse ActRIIA cDNA, subcloned into pcDNA3, was generously provided by Dr. K. Miyazono (The JFCR Cancer Institute). Human BMPRII and partial mouse ActRIIB cDNA clones were obtained from ATCC. BMPRII cDNA construct was tagged with a C-terminal HIS tag and subcloned into the pCAGGS vector. A full length flag-tagged ActRIIB cDNA was obtained by RT-PCR from adult mouse brain and subcloned into the pCMVTag4 vector (Stratagene).

### Chemotaxis Assays

The WEHI 274.1 mouse monocytic cell line and the THP-1 human leukemia cell line (ATCC) were maintained in standard growth medium (DMEM/10% FBS/1x P/S/G). Cells were washed twice in nonsupplemented DMEM medium, counted using a hemocytometer, resuspended to 2×10^6^ cells/ml in serum-free medium and pre-incubated for 1 hour at 37°C. For PI3K inhibitor experiments, 50 µM LY294002 (Cell Signaling Technology) or 100 nM Wortmannin (Sigma) was added to the cells during the pre-incubation period. WEHI 274.1 cells (100 µl) were then added to the upper chamber of 24-well, 5 µm transwell filter inserts (Costar) and 600 µl of recombinant BMP or MCP-1 diluted in nonsupplemented DMEM added to the lower chamber. BMPs have been shown to be potent chemotactic agents with maximal stimulation of human monocytes around 0.1–1 pg/ml (3–30 fM) [7], [8]. Our dose response curve confirms these findings and for routine experiments 1 pg/ml or 10 pg/ml was used. After 30 minutes at 37°C, the filter inserts were washed in PBS, fixed with 2.5% gluteraldehyde (Sigma) and stained with Gill's Hemotoxylin (Sigma) as per the manufacturer's instructions. The filters were mounted onto glass microscope slides in 50% glycerol and coverslipped. Cells within the filter pores were counted from 4 fields (viewed at 20x) each from duplicate or triplicate filters (1000–3000 cells/filter) using a Zeiss Axiovert inverted microscope. Results are presented as the chemotaxis index (CI)  =  ((# treated cells in filter pores) − (# control cells in filter pores)/(# control cells in filter pores))×100 (mean +/− SEM).

Zigmond-Hirsh checkerboard analysis was used to assess the chemotactic versus chemokinetic response of WEHI 274.1 cell migration to BMP7 by varying the concentration of BMP7 in both the upper and lower transwell chambers [52].

### RT-PCR for Type II BMP Receptors

Primers for the 3 mouse type II receptor subunits (ActRIIA, ActRIIB, and BMPRII) were used as previously described [53]. Total RNA (Trizol Reagent, Invitrogen) was prepared from suspension cultures of WEHI 274.1 cells. Reverse transcription of RNA (2 µg) was performed using oligo dT primers and Superscript II reverse transcriptase (Invitrogen). Amplification of individual type II BMP receptor fragments was performed for each primer set using 5 Prime MasterMix (5 Prime, Inc.).

### Design and Construction of shRNA Vectors

For RNAi-mediated down-regulation of type II BMP receptor subunits, synthetic oligonucleotides were designed to target sequences 100% conserved in human, mouse and rat *ActRIIA, BMPRII* and *Smad4*, using shRNA design guidelines [54] and the RNAi design program at the Whitehead institute (http://jura.wi.mit.edu). Oligonucleotides were annealed and ligated into the LentiLox3.7 lentiviral vector (pLL3.7, generously provided by Dr. L. Van Parijs, MIT) under the control of the U6 promoter [55] and used to produce shRNAs for the specific targets. This plasmid also encoded EGFP under the control of the CMV promoter, effectively tagging all shRNA-expressing cells. Target sequences were as follows: ActRIIA (*sh-AIIA* - 5′ GCAGGAAGTTGTTGTGCAT 3′), BMPRII (*sh-BRII* - 5′ GGATGAGCGTCCAGTTGCT 3′) and Smad4 (*sh-Smad4* - 5′ ACAATGAGCTTGCATTCCA 3′). A lentiviral shRNA construct, targeting the mouse ActRIIB receptor (*sh-AIIB*), was obtained from Open Biosystems (clone ID# TRCN0000022641). Test transfections of the shRNA constructs were performed by Lipofectamine (Invitrogen) transfection into HEK 293 and COS-1 cells. *sh-dsRed*, targeting red fluorescent protein, was used as a functional negative control (*sh-dsRed* - 5′ GCAGCGTCGTTCGATACTA 3′). *sh-BRII*, *sh-Smad4*, and *sh-dsRed* constructs were generously provided by Dr. P. Scheiffele, Columbia University. *sh-AIIA*-resistant ActRIIA cDNAs were produced by site-directed mutagenesis of 1 or 3 Valine codons in the sequence targeted by *sh-AIIA*, ActRIIA-RES-1V (V431V) or ActRIIA-RES-3V (V431V, V432V, V433V), respectively, using the QuikChange Site-Directed Mutagenesis Kit (Stratagene).

### Electroporation and Cell Sorting

WEHI 274.1 cells were washed twice in nonsupplemented DMEM, added to a 4 mm electroporation cuvette (0.8 ml at 1.2×10^7^ cells/ml) with GFP-tagged shRNA vectors (20 µg), cDNA expression constructs (20 µg) or empty vector (20 µg) for control experiments and incubated for 15 minutes at 37°C. Cells were electroporated at 340 V, 960 µF using a BioRad GenePulser. Cells were left to recover (10 minutes, 37°C), then plated in 20 ml fresh growth medium for 12 hours and washed. GFP positive cells were subjected to fluorescence-activated cell sorting (FACS) (Ultra Hypersort Flow Cytometer, Beckman Coulter). Sorted cells (^Δ^WEHI) were washed in fresh growth medium and plated for 12 hours before harvesting for chemotaxis assays and Western analyses.

### Quantitative PCR (QPCR)

One microgram of total RNA (Trizol Reagent, Invitrogen), isolated from sorted, shRNA-expressing WEHI 274.1 cells, was reverse-transcribed using RevertAid First Strand cDNA Synthesis Kit (Fermentas). For individual experiments (n = 2), three 20 µl cDNA reactions were prepared from each RNA sample and pooled. A single 20 µl cDNA reaction was prepared in the absence of reverse transcriptase (No RT control). Real-time QPCR was performed on the Mx3000P QPCR apparatus and analyzed on MxPro-Mx3000P v4.0 software (Stratagene). Serial dilutions of wild-type WEHI 274.1 cDNA were used to determine the amplification efficiency for each primer set (see Table 1) using 2X Maxima SYBR Green qPCR Master Mix (Fermentas). Reaction conditions were as follows: denaturation for 10 minutes at 95°C, followed by 40 cycles of 30 seconds at 95°C, 1 minute at 58°C, and 1 minute at 72°C. Comparative quantitative analysis was performed using β-actin as a normalizing control and expressed relative to BMP receptor mRNA levels in dsRed^Δ^WEHI cells. Three individual comparative analyses, which included No RT and No Template controls, were performed on each set of pooled cDNA.

**Table 1:** Primers for real-time quantitative PCR.

Target	Primers	Reference
ActRIIA	GTTGAACCTTGCTATGGTGATAA, AATCAGTCCTGTCATAGCAGTTG	[56]
ActRIIB	CACAAGCCTTCTATTGCCCACAG, CATGTACCGTCTGGTGCCAAC	[57]
BMPRII	TGGCAGTGAGGTCACTCAAG, TTGCGTTCATTCTGCATAGC	[58]
β-actin	TGCGTGACATCAAAGAGAAG, GATGCCACAGGATTCCATA	[59]

Sequences and literature references for primer sets used in QPCR.

### Western Blot Analysis

Whole cell lysates were prepared using 1x Lysis Buffer (Cell Signaling Technology). Samples were separated by SDS-PAGE (EZ-Run Gel Solution, Fisher Scientific) and transferred to nitrocellulose (Whatman). Nitrocellulose membranes were blocked in 5% non-fat milk/0.1% Tween 20/TBS (Blocking Buffer) and probed overnight with primary antibodies diluted in Blocking Buffer. Membranes were washed in TBST (0.1% Tween 20/TBS) and probed (1 hour) with HRP-conjugated secondary antibodies in Blocking Buffer. After washing in TBST, blots were developed using the Supersignal West Pico chemiluminescent substrate detection kit (Pierce). Densitometric analysis was performed using ImageJ 1.37v software (NIH).

For phosphorylation assays, WEHI 274.1 cells were stimulated with BMPs for 30 minutes before preparation of cell lysates. Membranes were blocked and the primary and secondary antibodies diluted in 5% non-fat milk/0.1% Tween 20/TBS, except for the phospho-Smad1/5/8 and phospho-Akt(S) antibodies which were diluted in 5% BSA/0.1% Tween 20/TBS.

### Northern Analysis

The C2C12 myoblast cell line (ATCC) was maintained in standard growth medium (DMEM/10% FBS/1x P/S/G). The cells were transfected with *sh-dsRed* or *sh-Smad4* using Lipofectamine Reagent (Invitrogen). Following transfection, the cells were incubated overnight in serum-free OptiMEM supplemented with 1x P/S/G. Cells were then stimulated with 150 ng/ml BMP7 for 1 hour. Total RNA was extracted from each sample using Trizol Reagent (Invitrogen). Ten micrograms of total RNA was separated on a MOPS/formaldehyde gel, transferred to nylon membrane (GeneScreen Plus, Perkin Elmer) and hybridized with a digoxigenin-labeled RNA antisense probe, generated from a mouse Id1 cDNA construct (NIH Mammalian Gene Collection, Invitrogen). Digoxigenin labeling of the probe, hybridization, and chemiluminescent detection were performed using the DIG Northern Starter Kit and the DIG Wash and Block Buffer Set (Roche). BMPs have been shown to be potent chemotactic agents with maximal stimulation of human monocytes around 0.1–1 pg/ml (3–30 fM) [7], [8]. In contrast, BMP concentrations required to stimulate Smad phosphorylation or induce gene expression are considerably higher [7], [11].

## Supporting Information

Figure S1 THP-1 cell migration through transwell chamber filters in response to 10 pg/ml BMP7, BMP2, BMP6 or GDF7 in the lower chamber. For comparison, cells were also stimulated with MCP-1 (100 ng/ml). Results are presented as the chemotaxis index (CI)  =  ((# treated cells in filter pores) − (# control cells in filter pores)/(# control cells in filter pores))×100 (mean +/− SEM). MCP-1 (CI = 71+/−18, n = 3); BMP7 (CI = 125+/−31, n = 4); BMP2 (CI = 89+/−21, n = 3); BMP6 (CI = 4+/−17, n = 2); GDF7 (CI = 6+/−10, n = 2).
(0.14 MB TIF)

Figure S2 A: Stage 10 chick [1] intermediate spinal cord explants were isolated and cultured in three-dimensional collagen gels in OptiMEM medium supplemented with 1x Penicillin/Streptomycin/Glutamine (Invitrogen) as previously described [2]. Explants were incubated in control medium (No Rx) or 10 ng/ml BMP7, BMP6 or GDF7 (R&D Systems) for 48 hours, fixed in 4% paraformaldehyde (Electron Microscopy Sciences) and labeled with a rabbit α-LH2 (L1) antibody and a Cy3-conjugated goat-α-rabbit secondary antibody (Jackson Labs). Z-stack images were obtained on a Zeiss LSM510 confocal microscope. BMP7, BMP6 and GDF7 induced similar levels of LH2 expression. B: Whole cell lysates of WEHI 274.1 cells incubated with or without 50 ng/ml BMP7, BMP6 or GDF7 were probed on Western blots with a phospho-specific α-Smad1/5/8 antibody. GAPDH expression served as a loading control. Results are expressed as the percent of control (mean +/− SEM) for each condition relative to pSmad levels in control (No Rx) cells (n = 3). BMP7, BMP6 and GDF7 stimulated the phosphorylation of R-Smads to similar levels (∼40% over control). References: [1] Hamburger V, Hamilton H (1951) A series of normal stages in the development of chick embryo. J Morphol 88: 49–92. [2] Yamada T, Placzek M, Tanaka H, Dodd J, Jessell TM (1991) Control of cell pattern in the developing nervous system: polarizing activity of the floor plate and notochord. Cell 64: 635–647.
(0.68 MB TIF)

Figure S3 Western analysis of COS-1 whole cell lysates co-expressing control constructs and type II BMP receptor cDNA. A-C: Co-expression of ActRIIA (A), ActRIIB (B) or BMPRII (C) with *sh-dsRed* does not affect the level of receptor protein expression observed with empty vector (pLL3.7) co-expression. GAPDH expression served as a loading control.
(0.37 MB TIF)

Figure S4 Western analysis of whole cell lysates of HEK 293 cells, co-transfected with type II BMP receptor shRNAs or *sh-dsRed* and cDNA expression constructs, using α-ActRIIA, α-ActRIIB, or α-BMPRII antibodies, as indicated. These antibodies do not detect endogenous HEK 293 cell receptor protein, necessitating the use of a heterologous expression system to measure protein regulation in response to shRNA expression (J. C. Perron and J. Dodd, unpublished). Densitometric measurements of subunit bands were normalized to GFP or GAPDH and expressed relative to receptor expression levels in *sh-dsRed* transfected cells (mean +/− SEM, n = 3 for each condition). A–C: Relative ActRIIA protein levels in cells co-expressing ActRIIA cDNA and *sh-AIIA* (A), *sh-AIIB* (B) or *sh-BRII* (C). Quantitation, shown in histograms, shows that *sh-AIIA* inhibited ActRIIA expression by 90% (A). D–F: Relative ActRIIB protein levels in cells co-expressing ActRIIB cDNA and *sh-AIIA* (D), *sh-AIIB* (E) or *sh-BRII* (F). *sh-AIIB* inhibited ActRIIB expression by 45% (E). G–I: Relative BMPRII protein levels in cells co-expressing BMPRII cDNA and *sh-AIIA* (G), *sh-AIIB* (H) or *sh-BRII* (I). *sh-BRII* inhibited BMPRII expression by 80% (I). The type II BMP receptor shRNAs modulated target subunit protein selectively. Non-target BMP receptor expression was unchanged for all combinations with one exception: although *sh-AIIB* had no effect on BMPRII expression (H), expression of exogenous, flag-tagged mouse ActRIIA increased (B). These results were replicated using epitope tag antibodies to identify the individual heterologously expressed receptors (J. C. Perron and J. Dodd, unpublished).
(0.86 MB TIF)

Figure S5 dsRed^Δ^−, ActRIIA^Δ^−, ActRIIB^Δ^− and BMPRII^Δ^WEHI cells were tested for R-Smad phosphorylation in response to 50 ng/ml BMP7 for 30 minutes at 37°C. Whole cell lysates were probed on Western blots with a phospho-specific α-Smad1/5/8 antibody. Total Smad, GAPDH, and GFP expression served as loading controls.
(0.57 MB TIF)

Figure S6 Western analysis and quantitation of COS-1 cell co-transfections of *sh-dsRed* or *sh-AIIA* with wild-type ActRIIA cDNA and co-transfection of *sh-AIIA* with either ActRIIA-RES-1V or ActRIIA-RES-3V mutant cDNAs. ActRIIA-RES-1V and ActRIIA-RES-3V mutant cDNAs both showed resistance to the activity of *sh-AIIA*; ActRIIA-RES-1V (22% reduction) and ActRIIA-RES-3V (2% reduction) compared to wild-type ActRIIA (90% reduction) in the presence of *sh-AIIA*. Results are expressed as the percent of control (mean +/− SEM) for each condition relative to ActRIIA protein in *sh-dsRed*-expressing cells (control) cells (n = 3 for each condition). GFP expression served as a loading control.
(0.31 MB TIF)

Figure S7 A: Western blots of whole cell lysates of control and 100 nM Wortmannin (WM)-treated WEHI 274.1 cells, incubated with or without 50 ng/ml BMP7 for 30 minutes, were probed with a phospho-specific α-Smad1/5/8 antibody. Measurement of total Smad and GAPDH provided loading controls. Inhibition of PI3K activity did not affect BMP7-evoked stimulation of R-Smad phosphorylation. B: Chemotaxis of WEHI 274.1 cells in response to 10 pg/ml BMP7 was significantly reduced following 100 nM WM treatment (78% reduction). Results are expressed as the mean +/− SEM. BMP7-WM (CI = 56+/−7, n = 3) v. BMP7+WM (CI = 13+/−3, n = 3), p = 0.0042 (Student's t test). Treatment with WM alone (control conditions) had no effect on WEHI 274.1 cell movement (CI = 3.7+/−3.5, n = 2; J. C. Perron and J. Dodd, unpublished).
(0.33 MB TIF)

Table S1 Migration of WEHI 274.1 cells was measured in response to manipulation of BMP7 concentration gradients in transwell chemotaxis assays (value  =  Chemotaxis Index  =  ((# treated cells in filter pores) − (# control cells in filter pores)/(# control cells in filter pores))×100, mean +/− SEM, n≥3 for each condition). Distinct gradients (G) were generated by placing high and low concentrations of BMP7 in upper and/or lower chambers, as indicated (G =  [*x*:*y*], where *x* and *y* represent the concentration of BMP7 in the upper and lower chambers, respectively; *0*  =  No treatment, *1p*  =  1 pg/ml BMP7 and *100n*  =  100 ng/ml BMP7). Neutralization of the concentration gradient (bold numbers), by addition of equal concentrations of BMP7 to the upper and lower compartments (i.e. G =  [*1p:1p*] or G =  [*100n:100n*]), reduced the response to control levels. 1 pg/ml BMP7 in the lower chamber (G =  [*0:1p*]) significantly stimulated WEHI 274.1 cell migration towards the source of BMP7 compared to migration observed in the absence of a gradient of BMP7 (p = 0.00038 for [*0:1p*] v. [*1p:1p*]), demonstrating that BMP7 acts as a chemoattractant at this concentration. 1 pg/ml BMP7 in the upper chamber only (G =  [*1p:0*]) did not evoke migration through the filter (p = 0.626 for [*1p:0*] v. [*1p:1p*]), indicating that BMP7 does not simply increase baseline chemokinetic activity of WEHI 274.1 cells. The presence of high concentrations of BMP7 in the lower chamber (G =  [*0:100n*]) markedly inhibited WEHI 274.1 cell movement into the transwell filter compared to migration in the absence of a BMP7 gradient (p = 0.0075 for [*0:100n*] v. [*100n:100n*]). In contrast, migration towards the lower chamber was stimulated when 100 ng/ml BMP7 was added to the cells in the upper chamber (G =  [*100n:0]*) demonstrating a chemorepellent activity of BMP7 at high concentrations. The observed increase in chemotaxis in response to 100 ng/ml BMP7 in the upper chamber, however, was not quite significantly different from chemotaxis in the absence of a BMP7 gradient (p = 0.059 for [*100n:0*] v. [*100n:100n*]). Together, these results show that BMP7 stimulates chemotaxis in WEHI 274.1 monocytic cells, the direction of which is dependent on the BMP concentration. ND  =  Not Determined.
(0.03 MB DOC)
