# Supplementary figures and images for: ActRIIA and BMPRII Type II BMP Receptor Subunits Selectively Required for Smad4-Independent BMP7-Evoked Chemotaxis
Source: PLoS One. 2009 Dec 8;4(12):e8198. doi: 10.1371/journal.pone.0008198 (PMC2788225; doi:10.1371/journal.pone.0008198)

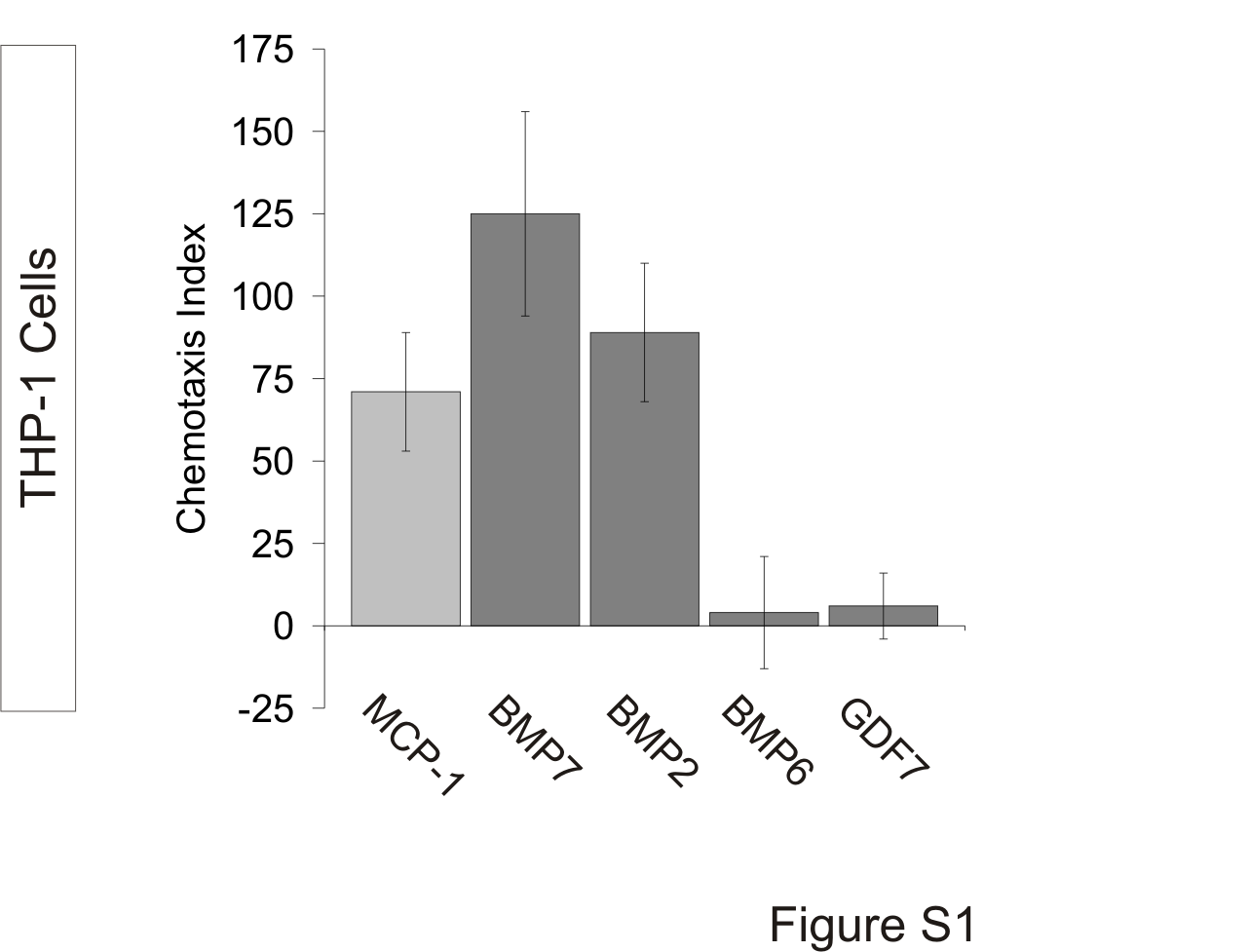

Supplement: Figure S1 — THP-1 cell migration through transwell chamber filters in response to 10 pg/ml BMP7, BMP2, BMP6 or GDF7 in the lower chamber. For comparison, cells were also stimulated with MCP-1 (100 ng/ml). Results are presented as the chemotaxis index (CI) = ((# treated cells in filter pores) − (# control cells in filter pores)/(# control cells in filter pores))×100 (mean +/− SEM). MCP-1 (CI = 71+/−18, n = 3); BMP7 (CI = 125+/−31, n = 4); BMP2 (CI = 89+/−21, n = 3); BMP6 (CI = 4+/−17, n = 2); GDF7 (CI = 6+/−10, n = 2). (0.14 MB TIF) [file pone.0008198.s001.tif]

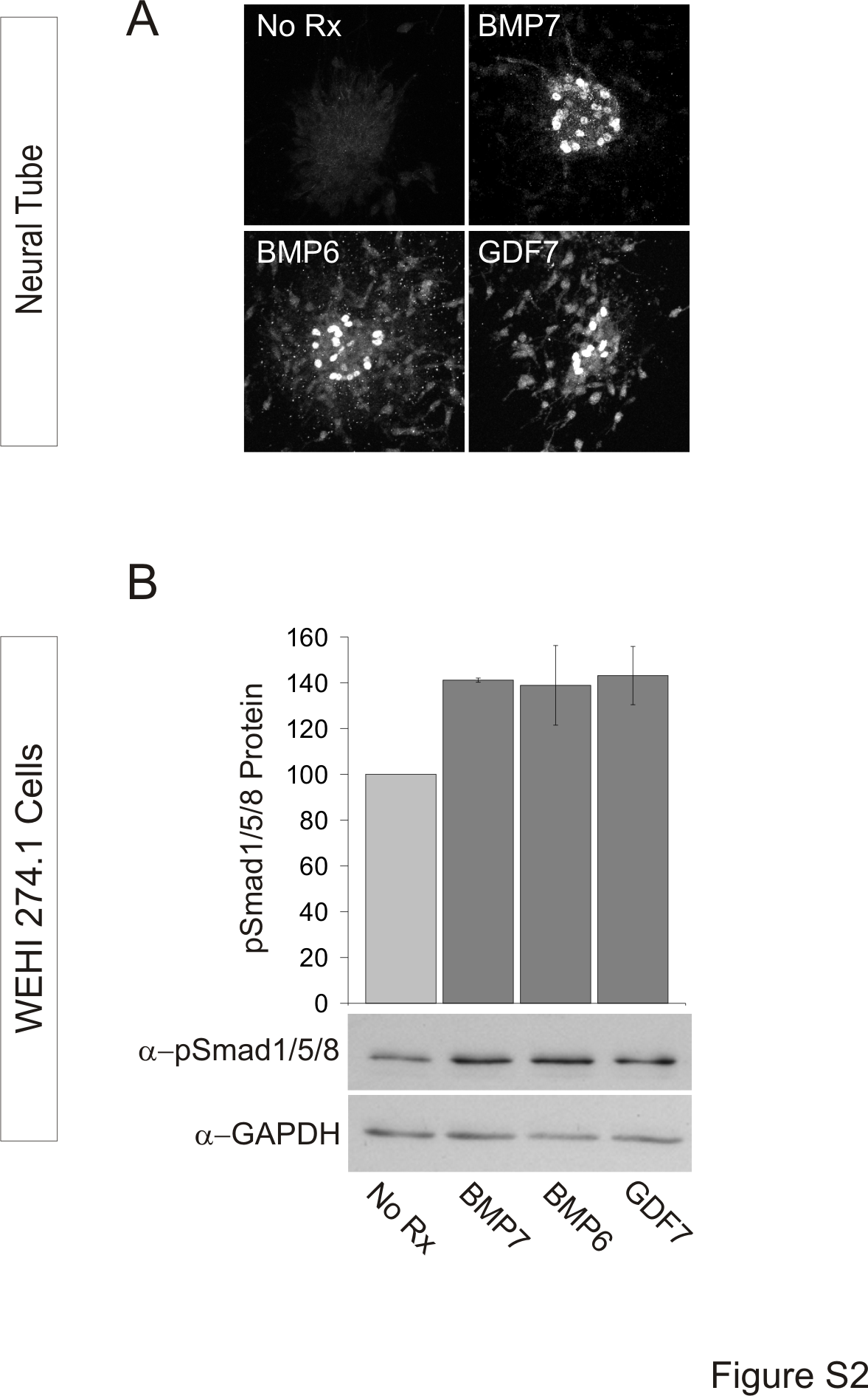

Supplement: Figure S2 — A: Stage 10 chick [1] intermediate spinal cord explants were isolated and cultured in three-dimensional collagen gels in OptiMEM medium supplemented with 1x Penicillin/Streptomycin/Glutamine (Invitrogen) as previously described [2]. Explants were incubated in control medium (No Rx) or 10 ng/ml BMP7, BMP6 or GDF7 (R&D Systems) for 48 hours, fixed in 4% paraformaldehyde (Electron Microscopy Sciences) and labeled with a rabbit α-LH2 (L1) antibody and a Cy3-conjugated goat-α-rabbit secondary antibody (Jackson Labs). Z-stack images were obtained on a Zeiss LSM510 confocal microscope. BMP7, BMP6 and GDF7 induced similar levels of LH2 expression. B: Whole cell lysates of WEHI 274.1 cells incubated with or without 50 ng/ml BMP7, BMP6 or GDF7 were probed on Western blots with a phospho-specific α-Smad1/5/8 antibody. GAPDH expression served as a loading control. Results are expressed as the percent of control (mean +/− SEM) for each condition relative to pSmad levels in control (No Rx) cells (n = 3). BMP7, BMP6 and GDF7 stimulated the phosphorylation of R-Smads to similar levels (∼40% over control). References: [1] Hamburger V, Hamilton H (1951) A series of normal stages in the development of chick embryo. J Morphol 88: 49–92. [2] Yamada T, Placzek M, Tanaka H, Dodd J, Jessell TM (1991) Control of cell pattern in the developing nervous system: polarizing activity of the floor plate and notochord. Cell 64: 635–647. (0.68 MB TIF) [file pone.0008198.s002.tif]

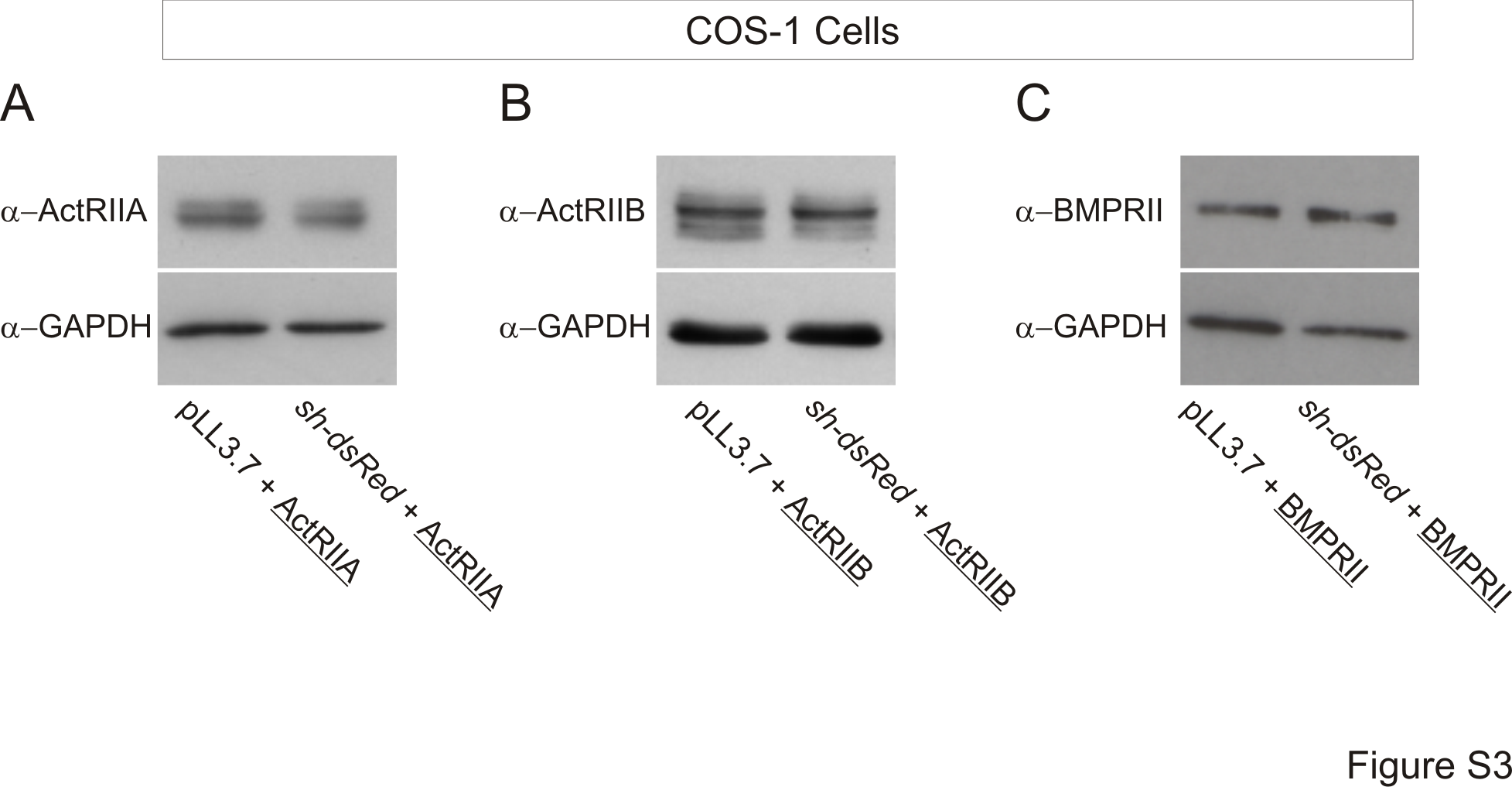

Supplement: Figure S3 — Western analysis of COS-1 whole cell lysates co-expressing control constructs and type II BMP receptor cDNA. A-C: Co-expression of ActRIIA (A), ActRIIB (B) or BMPRII (C) with sh-dsRed does not affect the level of receptor protein expression observed with empty vector (pLL3.7) co-expression. GAPDH expression served as a loading control. (0.37 MB TIF) [file pone.0008198.s003.tif]

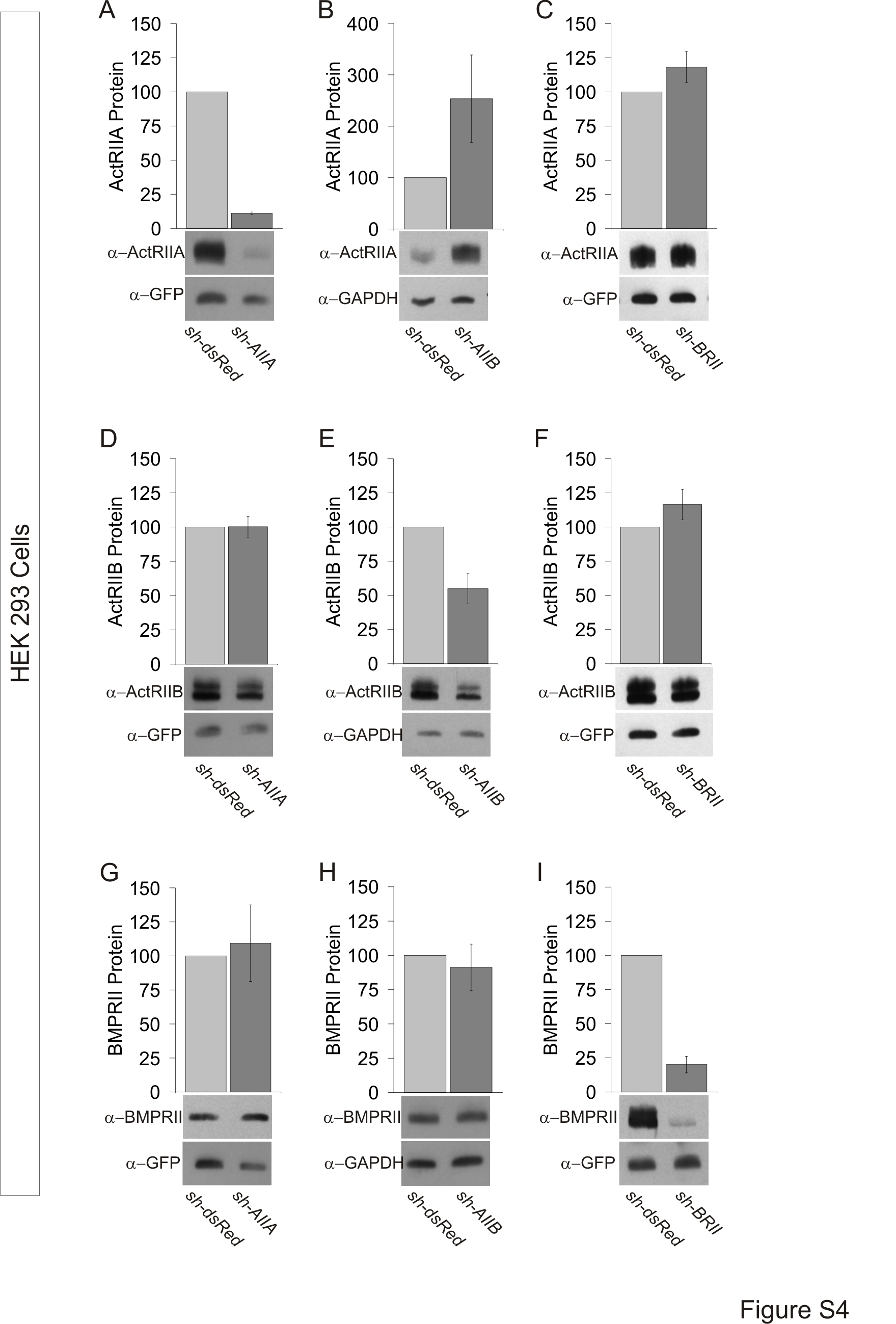

Supplement: Figure S4 — Western analysis of whole cell lysates of HEK 293 cells, co-transfected with type II BMP receptor shRNAs or sh-dsRed and cDNA expression constructs, using α-ActRIIA, α-ActRIIB, or α-BMPRII antibodies, as indicated. These antibodies do not detect endogenous HEK 293 cell receptor protein, necessitating the use of a heterologous expression system to measure protein regulation in response to shRNA expression (J. C. Perron and J. Dodd, unpublished). Densitometric measurements of subunit bands were normalized to GFP or GAPDH and expressed relative to receptor expression levels in sh-dsRed transfected cells (mean +/− SEM, n = 3 for each condition). A–C: Relative ActRIIA protein levels in cells co-expressing ActRIIA cDNA and sh-AIIA (A), sh-AIIB (B) or sh-BRII (C). Quantitation, shown in histograms, shows that sh-AIIA inhibited ActRIIA expression by 90% (A). D–F: Relative ActRIIB protein levels in cells co-expressing ActRIIB cDNA and sh-AIIA (D), sh-AIIB (E) or sh-BRII (F). sh-AIIB inhibited ActRIIB expression by 45% (E). G–I: Relative BMPRII protein levels in cells co-expressing BMPRII cDNA and sh-AIIA (G), sh-AIIB (H) or sh-BRII (I). sh-BRII inhibited BMPRII expression by 80% (I). The type II BMP receptor shRNAs modulated target subunit protein selectively. Non-target BMP receptor expression was unchanged for all combinations with one exception: although sh-AIIB had no effect on BMPRII expression (H), expression of exogenous, flag-tagged mouse ActRIIA increased (B). These results were replicated using epitope tag antibodies to identify the individual heterologously expressed receptors (J. C. Perron and J. Dodd, unpublished). (0.86 MB TIF) [file pone.0008198.s004.tif]

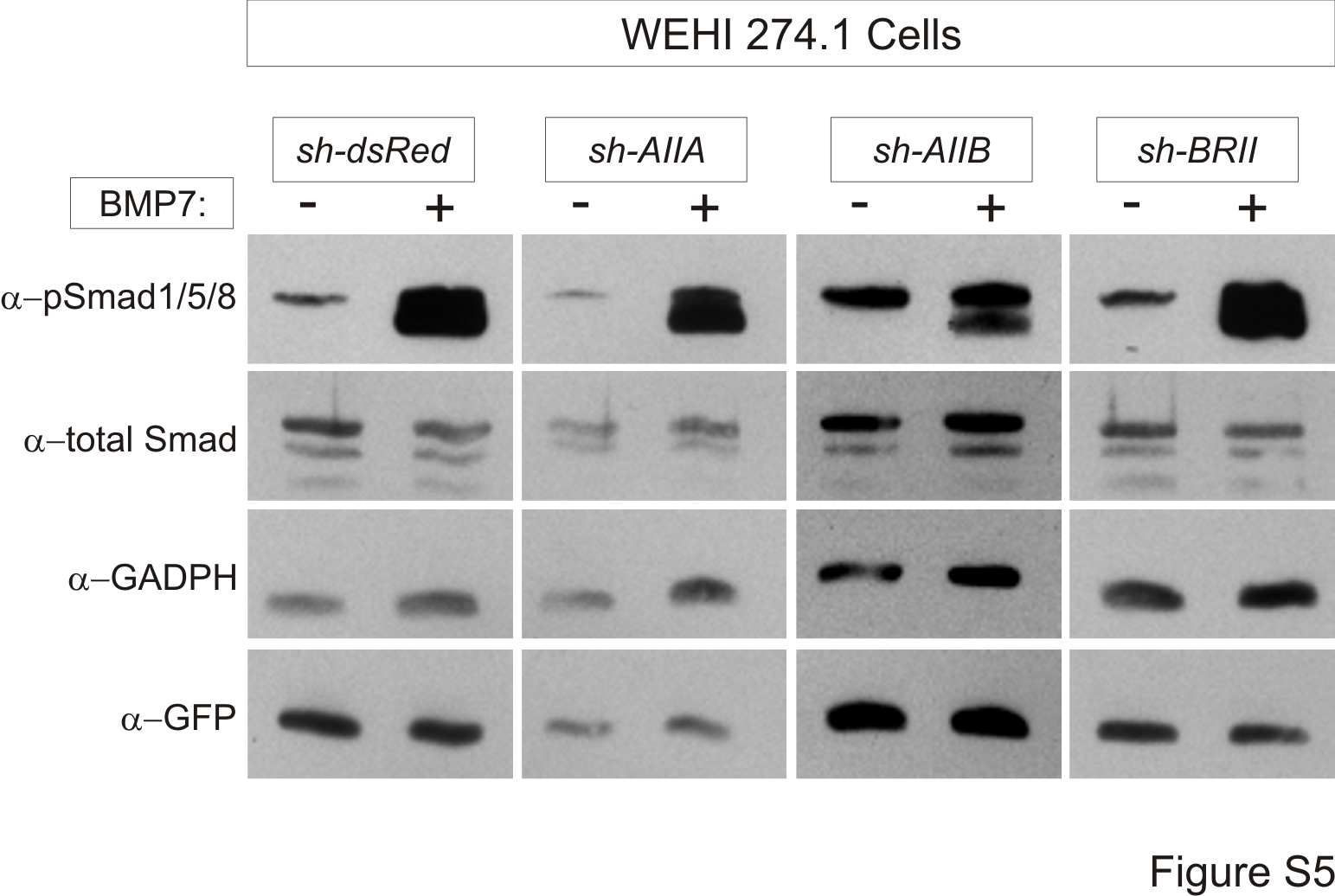

Supplement: Figure S5 — dsRedΔ−, ActRIIAΔ−, ActRIIBΔ− and BMPRIIΔWEHI cells were tested for R-Smad phosphorylation in response to 50 ng/ml BMP7 for 30 minutes at 37°C. Whole cell lysates were probed on Western blots with a phospho-specific α-Smad1/5/8 antibody. Total Smad, GAPDH, and GFP expression served as loading controls. (0.57 MB TIF) [file pone.0008198.s005.tif]

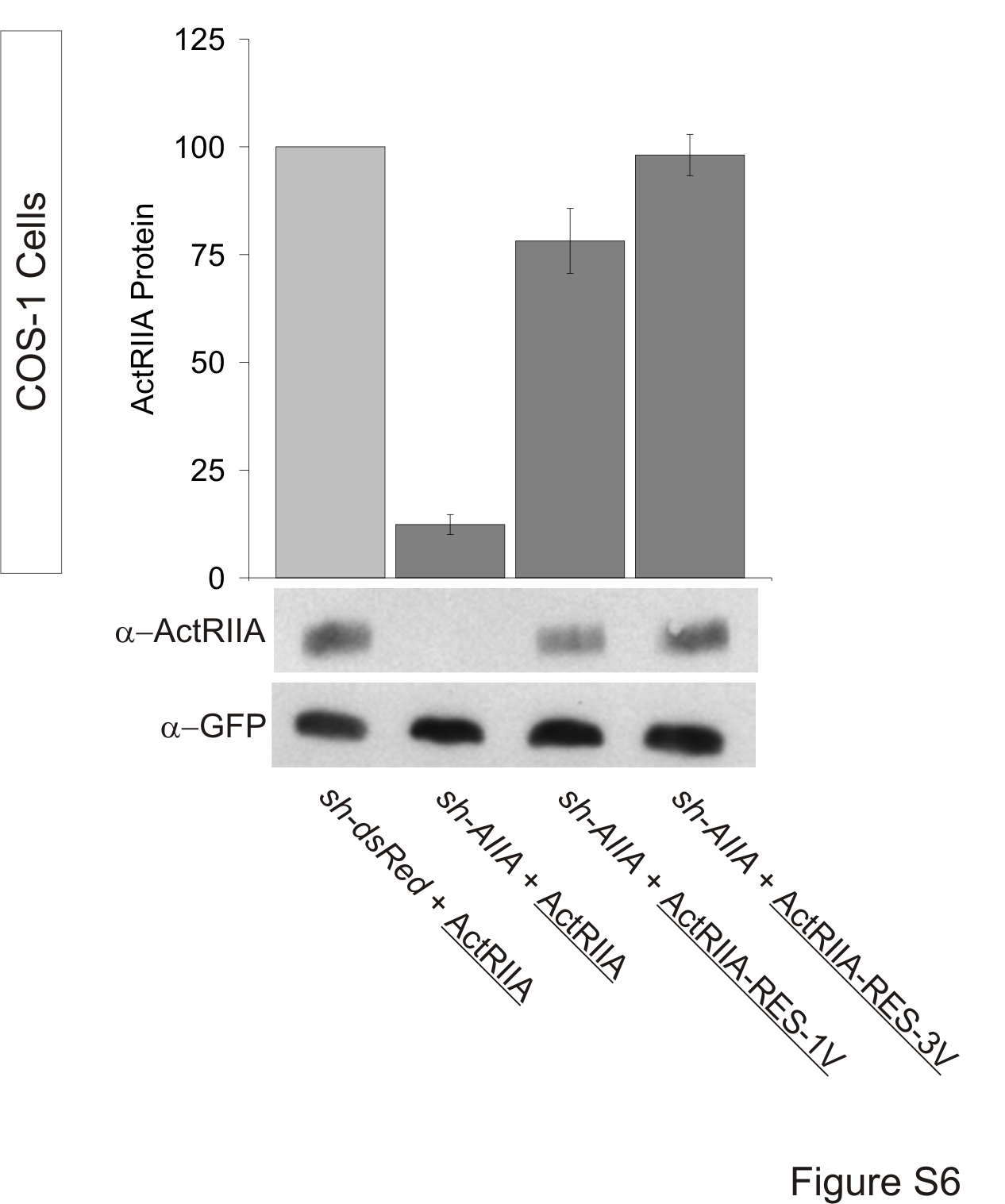

Supplement: Figure S6 — Western analysis and quantitation of COS-1 cell co-transfections of sh-dsRed or sh-AIIA with wild-type ActRIIA cDNA and co-transfection of sh-AIIA with either ActRIIA-RES-1V or ActRIIA-RES-3V mutant cDNAs. ActRIIA-RES-1V and ActRIIA-RES-3V mutant cDNAs both showed resistance to the activity of sh-AIIA; ActRIIA-RES-1V (22% reduction) and ActRIIA-RES-3V (2% reduction) compared to wild-type ActRIIA (90% reduction) in the presence of sh-AIIA. Results are expressed as the percent of control (mean +/− SEM) for each condition relative to ActRIIA protein in sh-dsRed-expressing cells (control) cells (n = 3 for each condition). GFP expression served as a loading control. (0.31 MB TIF) [file pone.0008198.s006.tif]

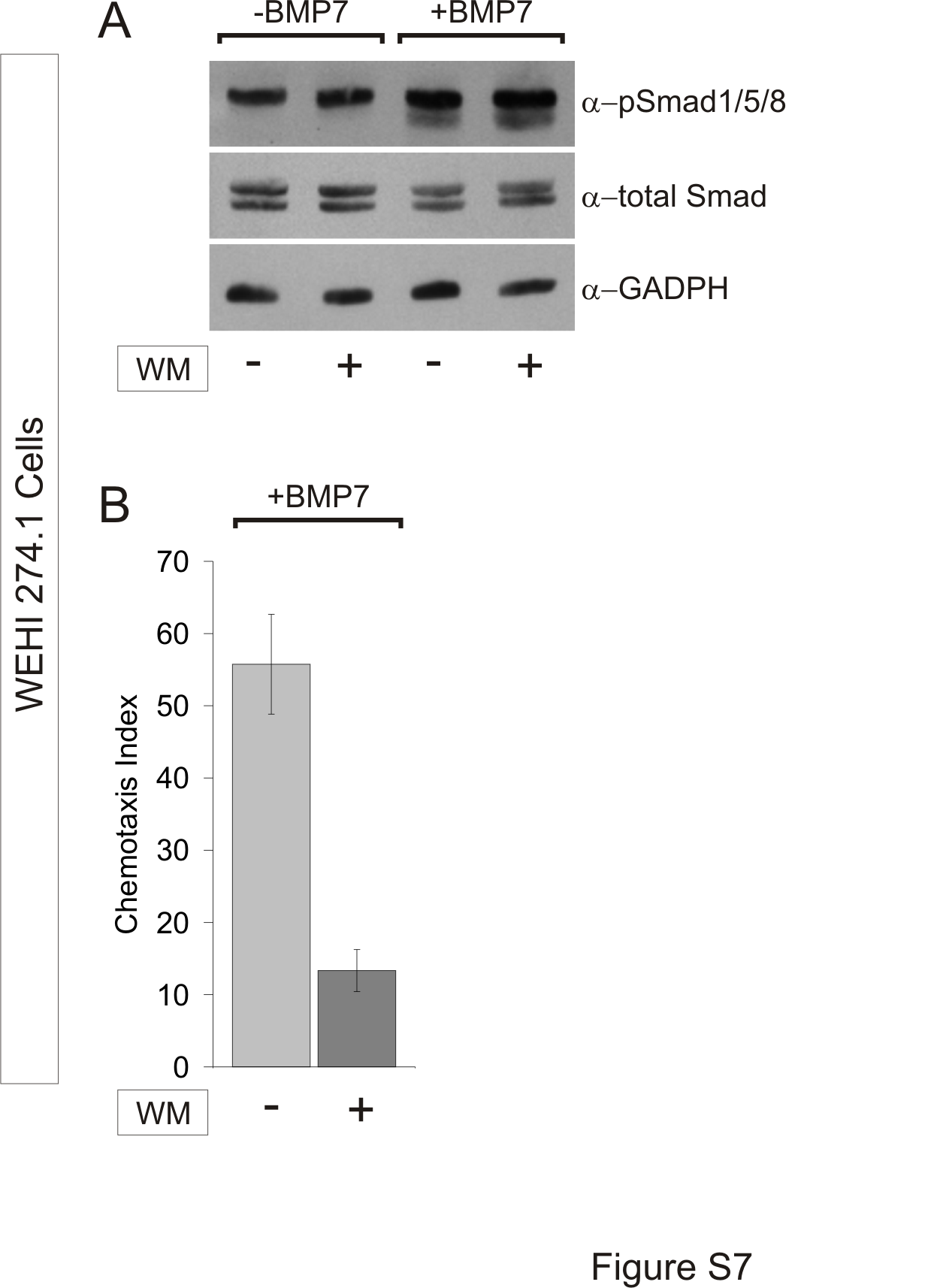

Supplement: Figure S7 — A: Western blots of whole cell lysates of control and 100 nM Wortmannin (WM)-treated WEHI 274.1 cells, incubated with or without 50 ng/ml BMP7 for 30 minutes, were probed with a phospho-specific α-Smad1/5/8 antibody. Measurement of total Smad and GAPDH provided loading controls. Inhibition of PI3K activity did not affect BMP7-evoked stimulation of R-Smad phosphorylation. B: Chemotaxis of WEHI 274.1 cells in response to 10 pg/ml BMP7 was significantly reduced following 100 nM WM treatment (78% reduction). Results are expressed as the mean +/− SEM. BMP7-WM (CI = 56+/−7, n = 3) v. BMP7+WM (CI = 13+/−3, n = 3), p = 0.0042 (Student's t test). Treatment with WM alone (control conditions) had no effect on WEHI 274.1 cell movement (CI = 3.7+/−3.5, n = 2; J. C. Perron and J. Dodd, unpublished). (0.33 MB TIF) [file pone.0008198.s007.tif]
