# Supplementary material for: ActRIIA and BMPRII Type II BMP Receptor Subunits Selectively Required for Smad4-Independent BMP7-Evoked Chemotaxis
Source: PLoS One. 2009 Dec 8;4(12):e8198. doi: 10.1371/journal.pone.0008198 (PMC2788225; doi:10.1371/journal.pone.0008198)
Supplement: Table S1 — Migration of WEHI 274.1 cells was measured in response to manipulation of BMP7 concentration gradients in transwell chemotaxis assays (value = Chemotaxis Index = ((# treated cells in filter pores) − (# control cells in filter pores)/(# control cells in filter pores))×100, mean +/− SEM, n≥3 for each condition). Distinct gradients (G) were generated by placing high and low concentrations of BMP7 in upper and/or lower chambers, as indicated (G = [x:y], where x and y represent the concentration of BMP7 in the upper and lower chambers, respectively; 0 = No treatment, 1p = 1 pg/ml BMP7 and 100n = 100 ng/ml BMP7). Neutralization of the concentration gradient (bold numbers), by addition of equal concentrations of BMP7 to the upper and lower compartments (i.e. G = [1p:1p] or G = [100n:100n]), reduced the response to control levels. 1 pg/ml BMP7 in the lower chamber (G = [0:1p]) significantly stimulated WEHI 274.1 cell migration towards the source of BMP7 compared to migration observed in the absence of a gradient of BMP7 (p = 0.00038 for [0:1p] v. [1p:1p]), demonstrating that BMP7 acts as a chemoattractant at this concentration. 1 pg/ml BMP7 in the upper chamber only (G = [1p:0]) did not evoke migration through the filter (p = 0.626 for [1p:0] v. [1p:1p]), indicating that BMP7 does not simply increase baseline chemokinetic activity of WEHI 274.1 cells. The presence of high concentrations of BMP7 in the lower chamber (G = [0:100n]) markedly inhibited WEHI 274.1 cell movement into the transwell filter compared to migration in the absence of a BMP7 gradient (p = 0.0075 for [0:100n] v. [100n:100n]). In contrast, migration towards the lower chamber was stimulated when 100 ng/ml BMP7 was added to the cells in the upper chamber (G = [100n:0]) demonstrating a chemorepellent activity of BMP7 at high concentrations. The observed increase in chemotaxis in response to 100 ng/ml BMP7 in the upper chamber, however, was not quite significantly different from chemotaxis in the absence of a [file pone.0008198.s008.doc]

**Table S1. Zigmond-Hirsh checkerboard analysis of directed migratory response to BMP7.**

|  | Lower Chamber | | | |
| --- | --- | --- | --- | --- |
| Upper Chamber | BMP7 | 0 | 1 pg/ml | 100 ng/ml |
|  | 0 0 | **0** | 236 +/- 21 | -54 +/- 0.5 |
|  | 1 pg/ml | 2 +/- 27 | **-5** +/- **13** | ND |
|  | 100 ng/ml | 119 +/- 27 | ND | **52 +/- 21** |

Migration of WEHI 274.1 cells was measured in response to manipulation of BMP7 concentration gradients in transwell chemotaxis assays (value = Chemotaxis Index = ((# treated cells in filter pores) – (# control cells in filter pores) / (# control cells in filter pores)) x 100, mean +/- SEM, n ≥ 3 for each condition). Distinct gradients (G) were generated by placing high and low concentrations of BMP7 in upper and/or lower chambers, as indicated (G= [*x:y*], where *x* and *y* represent the concentration of BMP7 in the upper and lower chambers, respectively; *0* = No treatment, *1p* = 1 pg/ml BMP7 and *100n* = 100 ng/ml BMP7). Neutralization of the concentration gradient (bold numbers), by addition of equal concentrations of BMP7 to the upper and lower compartments (i.e. G= [*1p:1p*] or G= [*100n:100n*]), reduced the response to control levels. 1 pg/ml BMP7 in the lower chamber (G= [*0:1p*]) significantly stimulated WEHI 274.1 cell migration towards the source of BMP7 compared to migration observed in the absence of a gradient of BMP7 (p= 0.00038 for [*0:1p*] v. [*1p:1p*]), demonstrating that BMP7 acts as a chemoattractant at this concentration. 1 pg/ml BMP7 in the upper chamber only (G= [*1p:0*]) did not evoke migration through the filter (p= 0.626 for [*1p:0*] v. [*1p:1p*]), indicating that BMP7 does not simply increase baseline chemokinetic activity of WEHI 274.1 cells. The presence of high concentrations of BMP7 in the lower chamber (G= [*0:100n*]) markedly inhibited WEHI 274.1 cell movement into the transwell filter compared to migration in the absence of a BMP7 gradient (p= 0.0075 for [*0:100n*] v. [*100n:100n*]). In contrast, migration towards the lower chamber was stimulated when 100 ng/ml BMP7 was added to the cells in the upper chamber (G= [*100n:0*]) demonstrating a chemorepellent activity of BMP7 at high concentrations. The observed increase in chemotaxis in response to 100 ng/ml BMP7 in the upper chamber, however, was not quite significantly different from chemotaxis in the absence of a BMP7 gradient (p= 0.059 for [*100n:0*] v. [*100n:100n*]). Together, these results show that BMP7 stimulates chemotaxis in WEHI 274.1 monocytic cells, the direction of which is dependent on the BMP concentration. ND = Not Determined.
